# Doublecortin undergo nucleocytoplasmic transport via the RanGTPase signaling to promote glioma progression

**DOI:** 10.1186/s12964-019-0485-5

**Published:** 2020-02-12

**Authors:** Abiola Abdulrahman Ayanlaja, Guanquan Ji, Jie Wang, Yue Gao, Bo Cheng, Kouminin Kanwore, Lin Zhang, Ye Xiong, Piniel Alphayo Kambey, Dianshuai Gao

**Affiliations:** 1 Department of Neurobiology and Anatomy, Key Laboratory of Neurobiology, Xuzhou Medical University, 209, Tongshan Road, Xuzhou, 221004 China; 2 Department of Neurosurgery, The Second Affiliated Hospital of Xuzhou Medical University, Xuzhou, Jiangsu China; 3 Department of Neurosurgery, The Third Affiliated Hospital of Henan University of Science and Technology, Henan, China

**Keywords:** Doublecortin (DCX), Glioblastoma Multiforme (GBM), RanGTPase pathway, Nucleocytoplasmic transport, Classical nuclear localization signals (cNLS)

## Abstract

**Background:**

Nuclear translocation of several oncogenic proteins have previously been reported, but neither the translocation of doublecortin (DCX) nor the mechanism involved has been studied. DCX is a neuronal microtubule-associated protein (MAP) that is crucial for adult neurogenesis and neuronal migration and has been associated with poor prognosis in gliomas.

**Methods:**

We probed DCX expression in different grades of glioma tissues and conventional cells via western blotting. Then we analyzed the expression pattern in the Oncomine cancer profiling database. Confocal Immunofluorescence was used to detect DCX expression in the cellular compartments, while subcellular fractionation was probed via western blotting. Pulse shape height analysis was utilized to verify DCX localization in a larger population of cells. Co-immunoprecipitation was used in detecting DCX-import receptors interactions. To probe for DCX functions, stable cells expressing high DCX expression or knockdown were generated using CRISPR-Cas9 viral transfection, while plasmid site-directed mutant constructs were used to validate putative nuclear localization sequence (NLS) predicted via conventional algorithms and comparison with classical NLSs. *in-silico* modeling was performed to validate DCX interactions with import receptors via the selected putative NLS. Effects of DCX high expression, knockdown, mutation, and/or deletion of putative NLS sites were probed via Boyden’s invasion assay and wound healing migration assays, and viability was detected by CCK8 assays in-vitro, while xenograft tumor model was performed in nude mice.

**Results:**

DCX undergoes nucleocytoplasmic movement via the RanGTPase signaling pathway with an NLS located on the N-terminus between serine47-tyrosine70. This translocation could be stimulated by MARK’s phosphorylation of the serine 47 residue flanking the NLS due to aberrant expression of glial cell line-derived neurotrophic factor (GDNF). High expression and nuclear accumulation of DCX improve invasive glioma abilities in-vitro and in-vivo. Moreover, knocking down or blocking DCX nuclear import attenuates invasiveness and proliferation of glioma cells.

**Conclusion:**

Collectively, this study highlights a remarkable phenomenon in glioma, hence revealing potential glioma dependencies on DCX expression, which is amenable to targeted therapy.

**Video abstract**

**Graphical Abstract:**

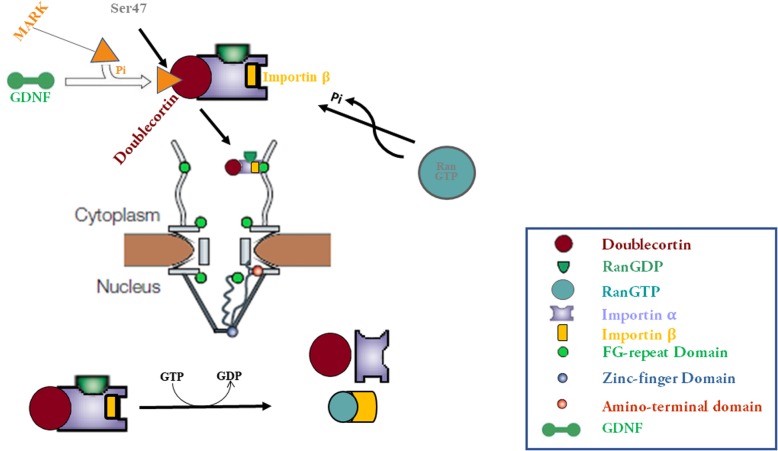

## Background

Although it would appear that all cancers require growth maintenance and survival of tumor cells, a series of molecular dysfunctions can produce similar outcomes, probably because of the complexity and redundancy of cellular signaling networks. The low efficiency of current conventional therapies may be due to the heterogeneous origin of malignant transformation in tumors [1]. Despite advances in new clinical therapies, the prognosis and survival of glioblastoma (GBM) patients remain dismal.

In recent years, a deeper understanding of different molecular mechanisms has led to many discoveries of the coordinated events regulating glioma development in favor of improved clinical diagnosis and treatment [2]. Among them are unusual trafficking of molecules into the nuclear compartment of gliomas, which can set a precedence for several chains of events that may lead to therapeutic resistance and recurrence. Nuclear localization of many oncogenic proteins is integral to their function in the same way that efficient subcellular trafficking of viruses/viral particles is crucial for pathogenicity [3]. There is increasing evidence that blocking the transport machinery of molecules into or out of the nuclear compartment may either attenuate or promote cancer progression [1]. It is also becoming increasingly apparent however, since specific cancer-related proteins, such as the tumor suppressors p53 [4], retinoblastoma protein (Rb) [5], the paracrine/autocrine signaling molecule parathyroid hormone-related protein (PTHrP) [3], CD44 [6], and EGFR [7], have been reported to transport through the nuclear pore to either activate or deactivate relevant signaling cascades. However, only a few pro-oncogenic molecules are known for nucleocytoplasmic movements.

Doublecortin (DCX) as a case study has never been reported to traffic the nuclear membrane. DCX is crucial for neuronal migration and differentiation of human neurons, and it is a reliable marker of adult neurogenesis due to its pivotal roles in neuroblast formation [8]. DCX is expressed in gliomas, and its contribution to the formation and migration of neuroblasts makes it susceptible to pro-oncogenic entities (reviewed in [9]). Rich et al. attributed the simultaneous expression of DCX with other cell migration regulators like osteonectin (SPARC) and semaphorin3B to tumor progression and poor patient survival [10]. DCX was also reported to be strongly expressed at the invasive front of gliomas regardless of the tissue grade or type [11], in patients-derived primary or recurrent GBM tumors [12], or cell lines [13]. Hence, it was proposed as a more specific marker of infiltrating gliomas [11]. However, despite these reports, Santra et al. frequently reported opposing views as they have also stated that glioma cells lack DCX, and they further proposed DCX is a tumor suppressor, as glioma progression was inhibited by induced DCX expression in their experiments [14–18]. Hence, further clarifications on DCX expression status and functions in gliomas needs to be explored. So far, little is known about the role of DCX in gliomas. It is generally understood to be localized in the cytosol due to its interactions with different cytoskeletal components and functions in stabilizing the cytoskeleton [19, 20].

Here, we clarified DCX expression in gliomas and report a RanGTPase-dependent nuclear import of DCX in glioma cells via a bipartite classical nuclear localization signal (cNLS). Furthermore, we explored the mechanism and consequences of DCX nuclear translocation and delineate its preponderance in glioma development. We also showed a nexus between DCX’s nuclear translocation and GBM invasive abilities.

## Materials and methods

Key resource table.

**Table:** 

**Antibodies**	**Source**	**Identifier (Cat/lot no.)**
β-Actin	Proteintech	Cat #; 66,009–1-lg
anti- GFAP	Abcam	ab53554 GR3224456–1
Vimentin	ProteinTech	Cat #60330–1-lg
anti- CD31	Abcam	ab28364
anti-EGFR	Abcam	ab32077
anti-Doublecortin	Abcam	ab207175
anti-FITC	Servicebio	SA00006–8
DAPI	Jiangsu KeyGEN BioTech	KGA215–10
Ran	Proteintech	I 0469–1-AP
Importin-α	Proteintech	18,137–1-AP
Importin-β	Abcam	GR287721–5
Mitofilin	Proteintech	10,179–1-AP
GAPDH	Proteintech	10,494–1-AP
Histone	Proteintech	GR312673–2
MARK1	Proteintech	21,552–1-AP
GDNF	Abcam	Ab207175
MARK1	Proteintech	21,552–1-AP
pDCX serine 297	Cell Signaling Technology	#4605
pDCX serine 334	Cell Signaling Technology	#3453

### Human tissue materials and ethics statement

Twenty-eight glioma and 10 non-neoplastic tissue samples from different patients (World Health Organization [WHO] Grades I-IV) were collected from The Affiliated Hospital of Xuzhou Medical University. Informed consent was obtained from patients under standard ethical procedures.

### Animals

Neonatal Sprague-Dawley (SD) rats within the ages of 2 to 3 days were obtained from the Experimental Animal Center of Xuzhou Medical University for rat’s astrocyte extraction. 30 male BALB/c nude mice (6 weeks old) were purchased from (Yangzhou University Center for Comparative Medicine) and were sacrificed according to established protocols by the National Institutes of Health Guide for the Care and Use of Laboratory Animals. All experimental procedures performed on animals were approved by the Animal Care Committee of Xuzhou Medical University.

### Cell culture and stem cell isolation

Human astrocytes-hippocampal cells (HA-h, Sciencell) were cultured in Astrocyte Medium (Sciencell). The human U251, U343, and U87 glioma cell lines, as well as rat’s C6 astroglioma cells, were purchased from National Infrastructure of Cell Line Resource, Shanghai. Human glioma cell lines and GV468 U251 were cultured separately in DMEM high glucose medium supplemented with 10% fetal bovine serum (FBS, Gibco) and penicillin-streptomycin, while C6 cells and GV468-C6 Cells were cultured in DME/F12 medium (Hyclone) supplemented with 10% FBS and penicillin-streptomycin. Neurospheres derived from C6 cell lines were cultured in DME/F12 medium without FBS, supplemented with 10 ng/ml epidermal growth factor (EGF), 10 ng/ml basic fibroblast growth factor (bFGF), and 20 ng/ml B27 supplement (PeproTech). Neurosphere cultures were maintained for 4–5 days before each passage with accutase. All cells were placed in similar conditions at 37 °C in a humidified 5% CO_2_ atmosphere. Western blots and immunofluorescence assays were performed to confirm the stem cells are nestin and CD133 positive.

### Rat astrocytes acquisition

Astrocytes were prepared from postnatal (days 1–3) SD rats, as previously described [21].

### DCX CRISPR-cas9 overexpression vector (GV468-DCX) and DCX cNLS plasmid construction and lentivirus packaging

#### DCX CRISPR-cas9 overexpression vector (GV468-DCX)

CRISPR-cas9 technology was used to generate cells stably expressing DCX overexpression or downregulation. Overexpression was carried out in rat C6 glioma cell line and human U251. The DCX gene fragment was prepared by polymerase chain reaction (PCR) amplification; upstream primer was 5 ‘-GGCGATCTGGTGGAGTTCGT-3 ‘, and the downstream primer was 5′-TTTGTTAGACGAAGCTT GGGCTGCA-3 ‘. The DNA fragment was ligated into the GV468 vector, and the recombinant plasmid was digested with GV468-DCX targeting at either human or rat DCX (Helper 1.0, Helper 2.0), transfected into 293 T cells to produce lentivirus, LV468, and an empty vector as control. Microscopic observation of green fluorescent protein GFP expression intensity was used to determine the efficiency of lentivirus infection while using virus gradient dilution method to determine the virus titer. The DCX mRNA and protein expression were detected by qRT-PCR and Western blot, respectively. Meanwhile, puromycin was used to screen cells expressing DCX mRNA and protein.

### DCX cNLS plasmid construction and lentivirus packaging

DCX gene cDNA library was used as a template to amplify and transform the DCX gene sequence to obtain DCX wild type vector and mutant I-IV: DCXNLS1-mut, DCXNLS1-del, DCXNLS2-mut, DCXNLS2-del; the eGFP-tagged DCX expression plasmids were constructed in pLenti-GIII-CMV-GFP-2A-Puro vector. After complete enzymatic digestion, the recovered digested product was retrieved using OMEGA’s E.Z.N.A.® Cycle-Pure Kit. Genes DCX vector, DCXNLS1-mut, DCXNLS1-del, DCXNLS2-mut, DCXNLS2-del were transferred from the pORF plasmid to pLenti-GIII-CMV-GFP-2A using the Fuzyme cloning kit (ABM Cat No. E044) Puro carrier. OMEGA’s E.Z.N.A.® Plasmid Mini Kit I was used to extract and digest plasmid DNA. Prior to transfection, 1.3–1.5 × 10^6^ ABM 293 T cells were transplanted into 10 cm cell culture dish in advance and cultured. 70–80% fusion rate was achieved at the time of transfection.

**Table:** 

Gene	Primer sequence(5′-3′)
DCXNLS1 del	CS31818-FP: GCATCTAGAAATATGAGAGGGTCATCAACGAATGGACTTCCAAGTCCCACTCA CS31818-RP:CTATGAGTGGGACTTGGAAGTCCATTCGTTGATGACCCTCTCATATTTCTAG
DCXNLS1 mut	CS31816-FP1:ACGAGACAAAGCATCTAGAAATATGAGACTTCCAAGTCCCACTCATAGTG CS31816-RP1:AGTGAGCACTATGAGTGGGACTTGGAAGTCTCATATTTCTAGATGCTTTG CS31816-FP:GTTGTAAAACGACGGCCAGTCTTAAGCTCGG CS31816-RP:TCCATGCTTCCAATCTTTCTGGATCCATCAATAGTG
DCXNLS2 del	CS31819-FP:AGGCATTAAGTAATGAGAAGGCAGCCAAGGCAGTACGTTTCTACCGCAATGGGGAC CS31819-RP:CCATTGCGGTAGAAACGTACTGCCTTGGCTGCCTTCTCATTACTTAATGCCTGC
DCXNLS2 del	CS31817-FP:ACAGAACCAGAACCTTGCAGGCATTATTCTACCGCAATGGGGACCGTTAC CS31817-RP:GAAGTAACGGTCCCCATTGCGGTAGAATAATGCCTGCAAGGTTCTGGTTC

### Molecular simulation

DCX structure was built by Modeller [22]. The crystal structure of 1MJD was used as the template to construct the DCX 3D model. To optimize the target structure built from Modeller, 10 ns of molecule dynamics were conducted on target structure using AMBER99SB force fields of Gromacs software [23]. Importin subunits were constructed using the crystal structure of the PDB database or using Modeller software utilizing the crystal structure as the templates. Protein interactions between DCX structure and Importin subunits were analyzed using HDOCK server [24, 25], Docking results analysis for proteins interaction were prepared by the PyMOL Molecular Graphics System (version 0.99 Schrödinger, LLC) and the histidine residues were protonated at pH 6.5 using the PDB2PQR server 6 [26]. The electrostatic interactions between DCX and Importin subunits were performed by the Adaptive Poisson-Boltzmann Solver (APBS) plug-in of the PyMOL software.

### PULSA

PulSA was conducted according to the previous description by Toh et al. [27]. We plated 3 × 10^5^ cells and cultured for 24 h. For RanGTP inhibition, cells were treated with 10 mM of GTPγS (Abcam) or GDPβS (Sigma) for 30 mins or 1 h, and the control groups were untreated. Cells were trypsinized and suspended in total media containing 10% fetal bovine serum albumin, washed with PBS and incubated with DCX antibody, followed by incubation in serum-free media and 4% paraformaldehyde fixation, series of washing before permeabilization in 0.1% Triton X-100, blocking with goat serum before staining with secondary antibody (FITC), and DAPI staining. Stained cells were then suspended in 1%(w/v) BSA/PBS for flow cytometry. Cells were probed in BD LSRFortessa™ flow cytometer. FCS files were exported and analyzed in Microsoft EXCELand histograms were plotted and analyzed by GraphPad prism and SPSS.

### Implantation and live imaging and tissue immunostaining

As previously described by Pandya et al. [28]. Animals were anesthetized and placed in a stereotaxic frame (Stoelting). Coordinates used for stereotactic injection were 3 mm right lateral and 1 mm posterior to bregma, with an injection depth of 3.5 mm. 3 μL of 4 × 10^5^ cells/μL suspension of GFP-expressing C6 cells in PBS. Cells were cranially injected into 6-week old BALB/c nude mice. For intranasal administration of GDNF, mice were placed in the supine position as previously described by Hashizume et al., [29]. A total of 50 μl of 200 ng/ml GDNF (5 μl every 2 min) was administered via the alternating sides of the nasal cavity twice before sacrifice. Bioluminescence images were captured twice every week. Histological images were taken with OLYMPUS DP70 microscope. Immunostaining images were captured using a Zeiss LSM 510 confocal microscope.

### Real-time quantitative PCR

Total RNA extraction and RT-qPCR were conducted as previously described [30].

Primer Information:

**Table:** 

Target Gene	Upstream Primer	Downstream primer
MARK1	5’TTATCGGCCTGGTACAGCTCA3’	5’GTGGTCCGCTATAAGCAGCA3’
GAPDH	5’TGACTTCAACAGCGACACCCA3’	5’CACCCTGTTGCTGTAGCCAAA3’

### Nucleocytoplasmic protein extraction, co-Immunoprecipitation, and Western blotting

Cytoplasmic and nuclear protein extraction reagents (Beyotime) with PMSF were used for the extraction of nuclear and cytoplasmic fractionations as described by the manufacturer. For co-Immunoprecipitation, lysates were extracted with IP buffer and carried out as previously described [31].

### Immunofluorescence staining

Immunofluorescence stained images were acquired with an Olympus fv10i laser confocal microscope.

### Wound healing and Transwell invasion assay

Wound healing and transwell invasion assays were carried out as previously described [2].

### Densitometric quantification of western blot bands

Digitalized WB bands were quantified by measuring pixel numbers with an automated digitizing system (Image J Studio). The total measured pixels of each band was normalized to the corresponding loading control.

### Statistical analysis

Comparisons for differences in means (± SD) were assessed by independent samples t-test or one-way ANOVA and LSD using GraphPad Prism 7 and SPSS 16, or otherwise stated in the figure caption. A *p*-value of ≤0.05 was considered significant, * *P* ≤ 0.05, **P ≤ 0.01, ***P ≤ 0.001.

## Results

Doublecortin is expressed in both cytoplasmic and nuclear compartments of glioma cells, and its expression is consistent with glioma grades.

**Fig. 1:**
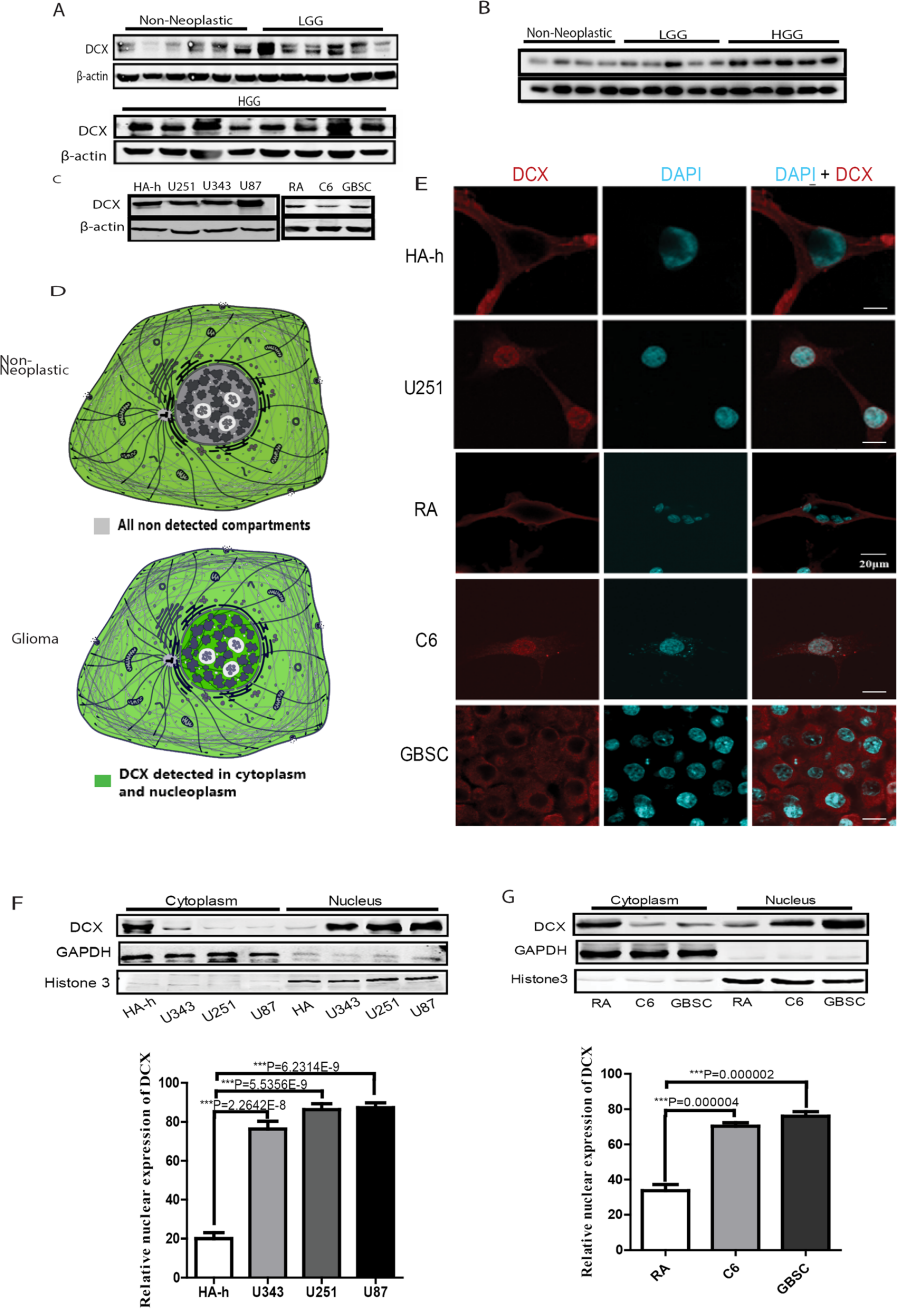
DCX is Expressed in both cytoplasmic and nuclear compartments of gliomas. **a** DCX protein levels as detected by western blotting in non-neoplastic tissues, LGG (low-grade gliomas patients), and HGG (WHO III&IV high-grade glioma). **b** Immunoblotting of another cohort excised at a different time and in a different set of patients derived tissues showing DCX protein expression. **c** DCX protein levels in human astrocyte-hippocampal (HA-h), U251MG, U343, U87MG, rat’s astrocytes (RA), C6 rat’s astroglioma cell line, and stem-like cells derived from C6 cells (GBSC), as detected by western blotting **d** Schematic drawing of DCX subcellular localization: general knowledge of DCX localization in normal cells (upper panel) and representative image of the current discovery of DCX subcellular localization in gliomas (lower panel) (image credit: Human Protein Atlas https://www.proteinatlas.org/ENSG00000077279-DCX/cell, https://www.proteinatlas.org/ENSG00000082898-XPO1/cell#gene_information). DCX-positive sites are marked green. **e** Subcellular localization of DCX (Red) in HA-h, U251, RA, C6, and GBSC as detected by immunofluorescence with representative laser confocal microscopy images. Scale bar, 20 μM. **f** Nucleocytosolic protein level of DCX as detected by western blotting and its subcellular localization quantitation. F = 300.715 and df = 3. **g** Western blotting and quantitation of DCX subcellular localization in RA, C6, and GBSC. F = 200.831 and df = 2

To clarify the conflicting reports on the expression and functions of DCX in gliomas [14–18, 32], we probed human clinical biopsies of WHO grades II (LGG), III and IV (HGG) including non-neoplastic tissue samples and conventional glioma cell lines of human origin (U251MG, U343, and U87) as well as human astrocytes-hippocampal (HA-h), or C6 astroglioma cells of rat origin and rat’s astrocytes (RA) for DCX protein expression. DCX expression was observed in grades II glioma tissues in the cohort of glioma samples probed with western blotting (WB) under similar condition, however, slightly higher than the non-neoplastic tissues, but significantly lower compared to high-grade gliomas (III and IV GBM tissues) (Fig. 1a). In another cohort excised at a different time and condition, and in a different set of glioma patients, immunoblotting showed that DCX expression in GBM tissues was higher than in low-grade gliomas and the non-neoplastic tissues (Fig. 1b). All human glioma cell lines probed showed preferential DCX expression relative to the respective astrocytic cells (Fig. 1c). Subsequent culture of C6 cells as neurospheres (glioblastoma stem-like cells, GBSC) suspended in DME/F12 medium supplemented with EGF, bFGF, and B27 supplement also indicated DCX expression (Fig. 1c). These results are consistent with reports from Rich et al. [10], and the expression profile of different brain tumor probed by Daou et al., [13]. To extend this, we analyzed datasets from the Oncomine cancer profiling database. A search for glioma datasets revealed that DCX is expressed in a wide range of glioma tissues from different datasets, and further analysis showed higher expression relative to normal tissues (Additional file 1: Figure S1a).

While probing for DCX fluorescence, we observed an irregular DCX expression in both the nuclear and cytoplasmic regions of C6, GBSC-C6, and U251MG cells, as opposed to the uniform distribution in the cytosol of both rat’s astrocytes and human astrocytes examined (Fig. 1e). To extend this, we probed for the subcellular localization of DCX. As detected by immunoblotting, the analysis revealed significantly higher DCX expression in the nucleus of U251, U87, U343, and rat’s astroglioma cells (C6 and GBSC) relative to their respective astrocytic cells (Fig. 1f and g). These data indicate that DCX is expressed in gliomas and can undergo nuclear translocation in glioma cells.

### Characterization of DCX nuclear translocation

**Fig. 2:**
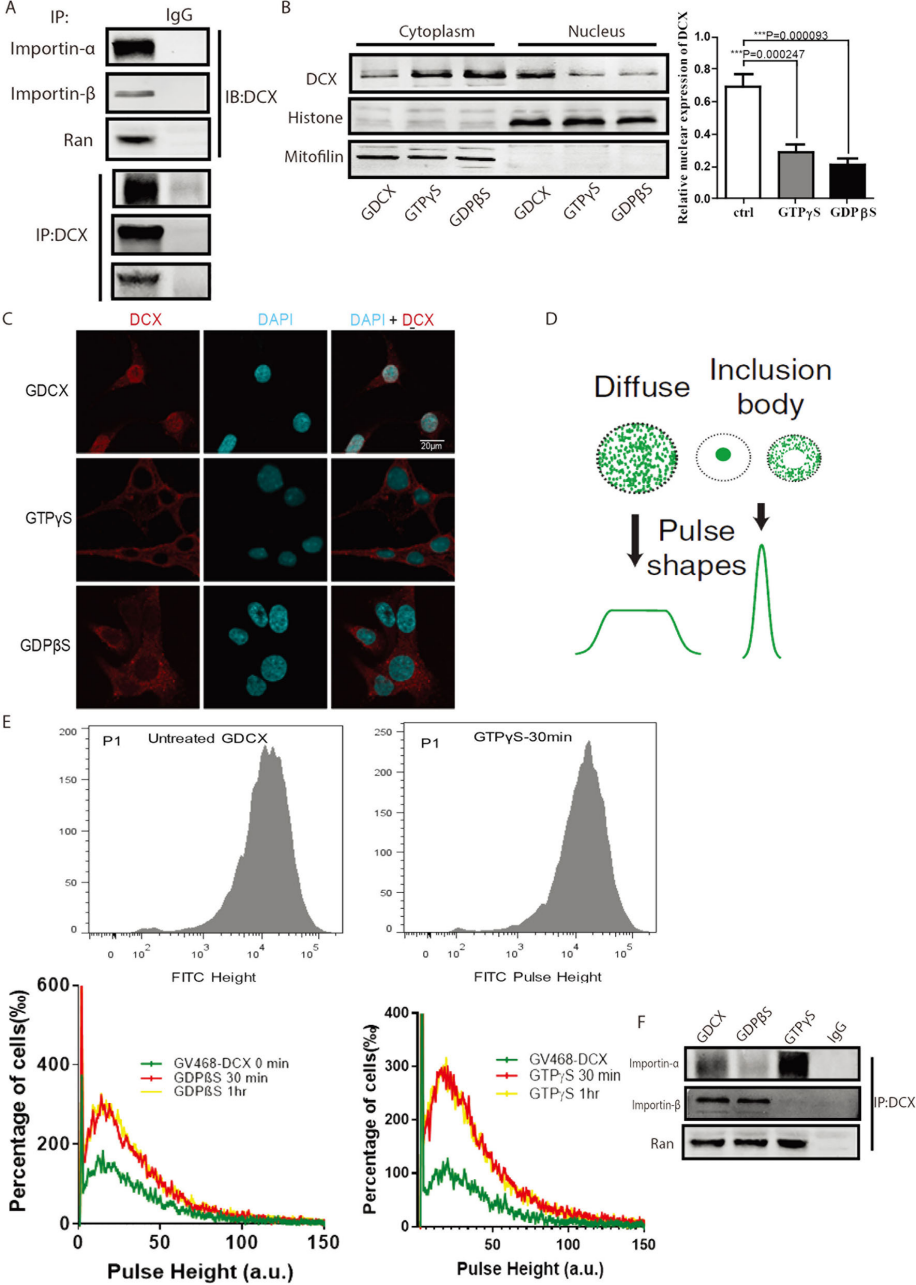
DCX nuclear import occurs via the RanGTPase dependent pathway. **a** Formation of protein-protein interaction of DCX with endogenous importin-α and importin-β by co-immunoprecipitation, Rabbit IgG was loaded in the second lane as the control immunoprecipitation. **b** Subcellular localization of DCX from GDCX-C6 cells treated with GTP analogs. F = 60.328 and df = 2. **c** Immunostaining of GDCX-C6 cells treated with GTPγS and GDPβS. Scale bar, 20 μM. **d** Schematics showing the principles of pulse shape analysis (PulSA), non-aggregated fluorescent protein generates a higher pulse height than inclusion body. **e** Cytograms showing pulse height of GTPγS-treated and untreated GDCX cells, upper panels. Histographs of analyzed data generated from flow cytometry experiments (in arbitrary units, a.u.), lower panels. Data are presented as Mean ± SEM. GDCX 0 min vs. GDPβS 30 min treatment (*P* = 0.0032, t = 2.964 df = 465.8), GDCX 0 min vs GDPβS 1 h treatment (*P* = 0.0031, t = 2.97 df = 541), GDCX 0 min vs GTPγS 30 mins treatment (*P* = 0.0001, t = 4.695 df = 882.6), GDCX 0 min vs GTPγS 1 h treatment (P = 0.0001, t = 4.595 df = 897). **f** Co-immunoprecipitation of DCX with importin-α and importin-β in whole-cell lysates of GTPγS, GDPβS, and untreated GDCX-C6 cells

Next, we sought to determine the mechanism involved in the translocation of DCX into the nuclear compartment. To determine the recognition of DCX by the nucleocytoplasmic transport receptors, we generated stable C6 and human U251 cells modified for high expression of DCX with GFP-fused CRISPR-Cas9 viral transfection to overexpress DCX (GDCX cells). For effective validation, we transfected dCas9 stable U251 and C6 cells with sgRNA targeted at human DCX or rat’s DCX, respectively. Co-Immunoprecipitation (Co-IP) assay carried out on lysates derived from GDCX-C6 cells for DCX against antibodies specific for importin-α5 (ProteinTech), and KPNB1/importin-β (Abcam) indicated that DCX binds to importin-α and importin-β, as well as Ran (Fig. 2a). We subsequently treated GDCX-C6 cells with 10 mM of G-protein-activating GTP analogs for 30 min; **γ-**glutamyltransferase **(**GTPγS, a non-hydrolyzable GTP analog that can preserve Ran in GTP-bound state**)** and **5**^**′**^**-** [β -thio] diphosphate trilithium salt (GDPβS, can restrict phosphorylation and keep Ran in an inactive GDP-bound state). Both treatments block GTPase activity as well as the GTP-GDP cycle [33]. Immunoblots indicated that both GTPγS and GDPβS significantly reduced the level of DCX in the nucleus of GDCX-C6 cells compared with untreated GDCX-C6 cells (Fig. 2b). Similarly, immunostaining indicated that both GTPγS and GDPβS abrogated DCX nuclear import in GDCX cells (Fig. 2c). These results imply that Ran in either the GTP- or GDP-bound state is crucial to DCX nucleocytoplasmic transport.

Pulse-shape analysis (PulSA) by flow cytometry has recently been utilized to monitor cargo trafficking between different intracellular compartments within a large population of cells [27] (Fig. 2d). Examination of height histogram of the whole population of cells revealed GTPγS- and GDPβS-treated GDCX-C6 cells had a higher pulse-shape than untreated GDCX cells (Fig. 2e). The change in pulse height signifies changes in the localization of DCX. This further indicates that disruption of the RanGTPase signaling pathway inhibits DCX translocation.

Next, we checked if the import receptors could still recognize DCX under the influence of these GTP analogs. GDPβS treatment abrogated binding between DCX and importin-α while GTPγS did not affect the binding of DCX to importin-α (Fig. 2f). This implies that the binding of DCX to importin-α may not necessarily require RanGTP but requires the RanGDP complex. Meanwhile, GTPγS restricted DCX-importin-β interactions, while GDP inhibition did not affect this bond (Fig. 2f). These data imply that Ran mediates the binding of DCX to importin β, and this is possible only in the presence of GTP; however, GDP may not contribute to these effects. Taken together, our functional analysis experiments indicate that DCX nuclear localization occurs via the RanGTPase signaling.

### Identification of DCX NLS

**Fig. 3:**
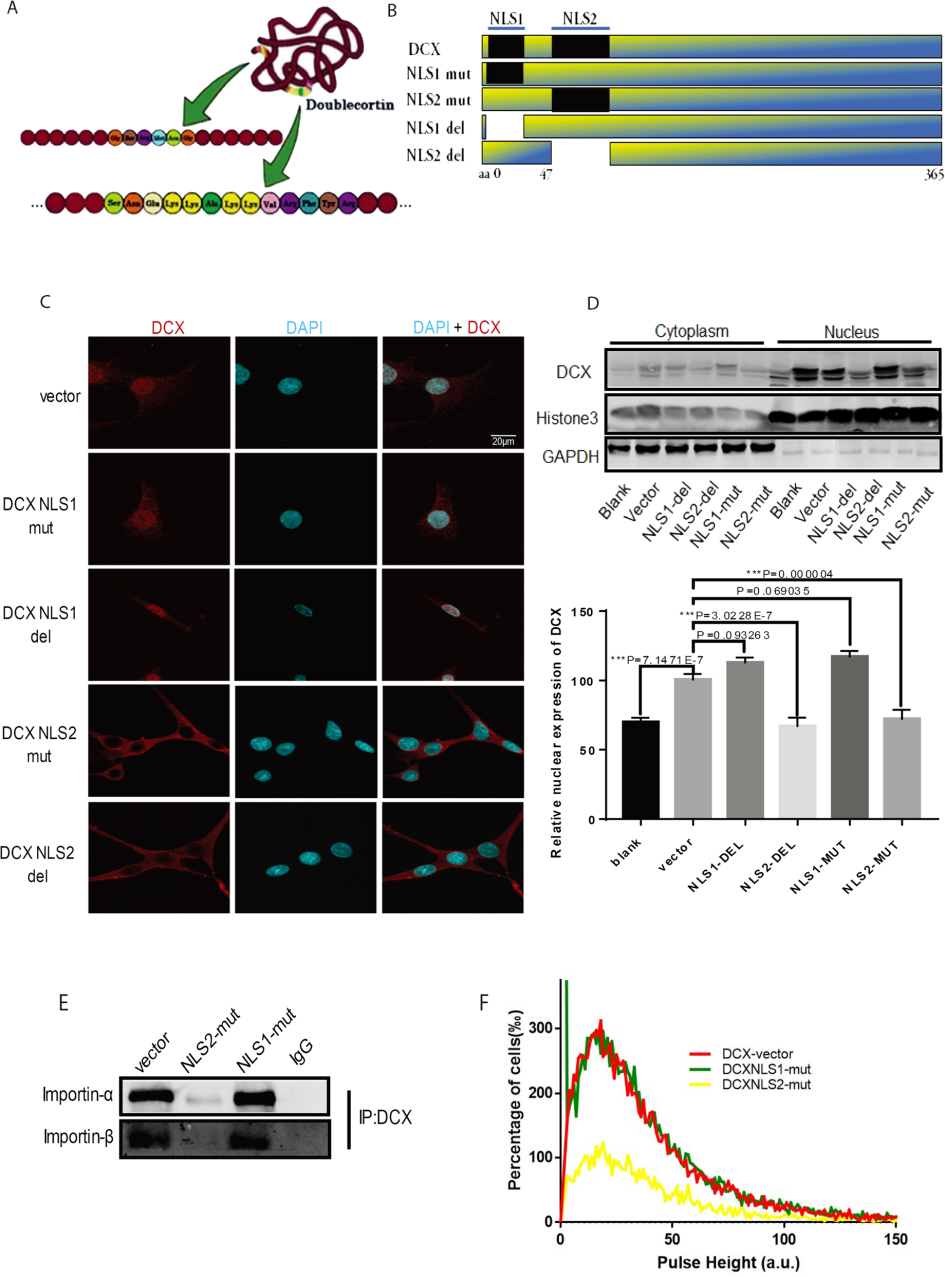
DCX nuclear import requires a classical nuclear localization signal. **a** Schematics of putative DCX NLS position and sequence as well as their structural position in the cartoon representation of DCX N-terminal region (suspension point indicates the extended part of DCX NLS2). **b** Illustration of DCX truncates containing putative DCX NLS mutants fused with GFP. Black bars indicate the location of NLS; blank spaces indicate deleted corresponding putative NLSs. **c** Subcellular localization of DCX in stable U251MG cells expressing DCX NLS mutant compared with DCX^+^ vector as detected by immunofluorescence. Scale bar 20 μm. **d** DCX subcellular localization levels in mutant cells as detected by western blotting. F = 67.156 and df = 5. **e** Co-immunoprecipitation of DCX with importin-α and importin-β in DCX mutants and vector control cells. **f** Pulse shape analysis of U251MG cells expressing DCX mutants compared with the vector showing lower pulse height in DCXNLS2 mutant cells

Most proteins that can translocate from the cytoplasm into the nuclear compartment possess sequence elements of either NLS, nuclear export signal (NES), or both. Since there was no known DCX NLS, we analyzed the amino acid sequence of DCX and found residues that resemble monopartite NLS (Fig. 3a). Conventional prediction algorithms like NLStradamus [34], SeqNLS/NLSUSC [35], and LOCTREE3 [36] predicted similar sequences around 20–25 and 47–69 on the N-terminus of DCX (Additional file 2: Figure S3a-c). Furthermore, we analyzed the DCX structure and function with the PREDICT PROTEIN database [37] and observed that the regions predicted by SeqNLS are binding sites on DCX, sites which may be where the importin complex binds to (Additional file 2: Figure S3d). Therefore, we selected “GSRMNG” as presented by seqNLS (tagged DCX-NLS1), and “SNEKKAKKVRFYRNGDRYFKGIVY” (tagged DCX-NLS2) based on the similarities to the classical SV40 NLS and resemblance to the bipartite Nucleoplasmin NLS [38].

For a putative NLS to be deemed a functional targeting sequence, it must be necessary for import, i.e., the transport of the protein of interest must be hindered when the sequence is altered [39]. Moreover, the mutation of a single residue of a functionally important motif can inactivate the functionality of an NLS [40]. Hence, we made mutations or deletions in DCX-NLS constructs in the context of full-length DCX (Fig. 3b, Additional file 6: Table S1). Immunostaining of stable U251 cells expressing these mutant vectors indicated diminished nuclear localization of DCX in both DCX^NLS2-mut^ and DCX^NLS2-del^ in contrast to the NLS1 constructs (Fig. 3c). Quantitation of the immunoblotting assays carried out on nuclear and cytosolic fractionations demonstrated consistent observation similar to those of the fluorescence assays (Fig. 3d). Co-IP assay showed diminished interaction between DCX and import receptors in DCX^NLS2-mut^, whereas DCX-importin-α/β interactions in DCX^NLS1-mut^ remained intact (Fig. 3e). As expected, analysis of the pulse height indicated lower DCX nuclear accumulation in the DCX^NLS2-mut^ cells compared with DCX^NLS1-mut^ (Fig. 3f). Overall, these results establish that DCXNLS2 is crucial for nuclear translocation of DCX in gliomas and may be sufficient for the chain of events leading to DCX translocation.

Furthermore, *in-silico* molecular docking between DCX and importin-α isoforms was performed with HDOCK software. We focused on the N-terminal region due to the DCX-NLS2 position in the context of full-length DCX, which is on the beta-sheet of the DCX structure. Both DCX NLS1 and NLS2 are located in an exposed loop as DCX NLS2 is flanked by an alpha helix in a computer-simulated human DCX N-terminal structure (1MJD.pdb) revealed by Modeller and pymol software (Additional file 2: Figure S3e). Our molecular docking results showed that all importin-α subunits docked successfully interacted with DCX at the NLS 2 region from Ser^**47**^ to Tyr^**70**^, indicating that residues ARG56, ARG 59, ARG 63, TYR64, LYS66, GLY 67, VAL 69, and TYR70 are key binding residues of DCX requiring 7–13 hydrogen bonds on each subunit for effective interactions (Additional file 2: Figure S3f-j).

### High DCX expression induces glioma proliferation and invasiveness

**Fig. 4:**
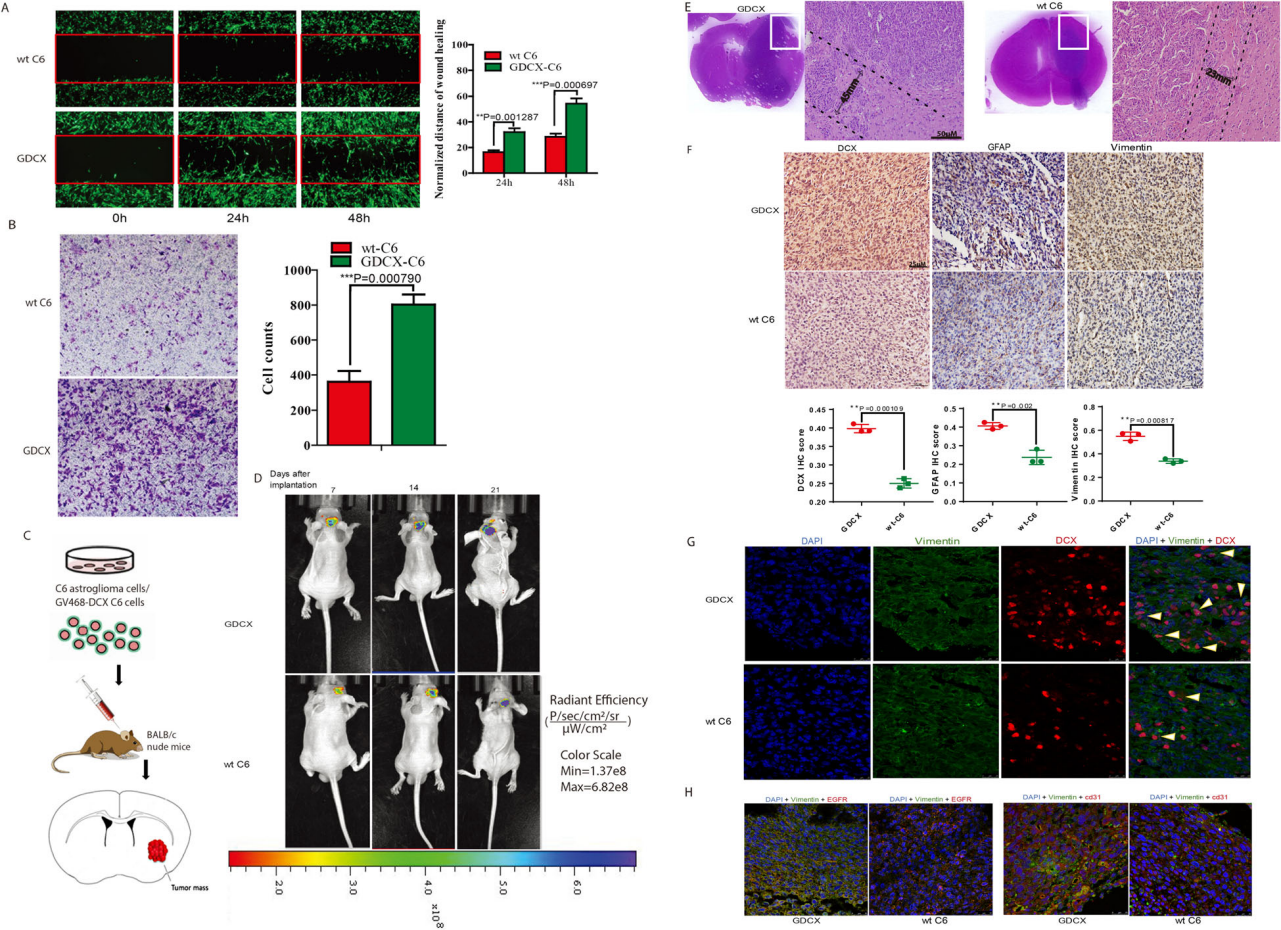
Induced High DCX Expression effectively improves GBM tumor growth and invasiveness. **a** Wound healing migration assay for detection of GFP-fused C6 (wt-C6) cell migration compared with GDCX-C6 cells, distance covered was quantified and represented by histogram data t = − 9.459 and df = 4. **b** Invasion assay conducted with GDCX-C6 and wt C6 cells and the quantification of invasive cells at the lower chamber. t = − 9.154 and df = 4. **c** Schematic of GDCX and wt C6 cells implantation into the brain of nude BALB/c mice. **d** Representative bioluminescence images from GDCX mouse and C6-GFP mouse. **e** Representative Hematoxylin-Eosin staining derived from of 8 μm tissue slices of GDCX and wt-C6 mice brain from a 200 μm region beyond the macroscopic boundary and the enlarged section of tumor edge showing infiltrating tumors at the invasive frontiers. The dashed boundary depicts the distance covered by invasive cells. Scale bar, 50 μm. **f** Immunohistochemical staining comparing DCX (df = 4, t = 15.211), GFAP (df = 4, t = 6.886), and vimentin (df = 4, t = 9.075) immunoreactivity in GDCX and wt-C6 tumors. Immunoreactivity was quantified using Image-Pro Plus, one to two sections per subject. Scale bars = 25 μm. **g** Immunostaining of brain sections showing DCX nuclear accumulation intensity with representative images of nuclei stained DAPI. Arrows indicate nuclear DCX-positive cells at the edge of tumors. Scale bars = 25 μm. **h** Representative immunofluorescence images of invasive glioma markers (CD31 and EGFR) in GDCX and wt-C6 tumors. Scale bar, 25 μm

The crucial roles of DCX in the development and migration of neuroblasts make it a vulnerable target for cancer cells (reviewed in [9]). However, detailed reports on the pro-oncogenic functions of DCX in glioma development remains exclusive. Given that DCX regulates neuroblast development during adult neurogenesis, we examined the relationship between high DCX expression and glioma progression. Stable cells expressing CRISPR-Cas9-induced DCX overexpression were utilized to probe the effects of high DCX expression on glioma cells. We observed that high DCX expression correlated with significantly enriched migration of GDCX-C6 cells via the wound healing migration assay (Fig. 4a). Similarly, Boyden chamber’s transwell invasion assay showed a relatively higher number of GDCX cells invaded the lower chamber compared with the wt-cells (Fig. 4b). Furthermore, significantly higher invasion of GDCX-C6 stem cells was observed when compared with the wt-C6 GBSCs (Additional file 3: Figure S4a). Next, we examined the effects of DCX on oncospheres formation which may reflect the self-renewal abilities of GBSCs. At day5, after induction, stem cells from GDCX and wt-C6 cells were compared by randomly counting spheres formed by each group on three separate fields. Additional file 3: Figure S4b indicated a significantly higher number of spheroids formed by GDCX-C6SC compared with wt-C6SC.

Furthermore, proliferation assays using the cell counting kit-8 revealed significant differences between the viability of GDCX cells and wt-C6 cells. Similarly, both GDCX non-stem like cells and stem cells showed a higher number of viable cells (Additional file 3: Figure S4c). To extend this, we subsequently transfected U251 cells with CRISPR-cas9 vectors to induce DCX-siRNA and compared viability. Our results indicated reduced viability in DCX-knockdown cells as detected by CCK8 assays (Additional file 3: Figure S4d), while re-expressing DCX with exogenous addition of recombinant DCX (150 ng/ul) in these cells improved the cell viability and proliferation (Additional file 3: Figure S4e). These results suggest that DCX promotes migration, invasion, as well as the proliferation of glioma cells in vitro.

Next, C6 cells and GDCX-C6 cells were injected intracranially in BALB/c nude mice brain (*n* = 12; 6 × 10^5^ cells per injection) to examine the influence of DCX in gliomas in vivo. Mice were monitored under similar conditions, and bioluminescence imaging was captured every week (Fig. 4d). Mice were then sacrificed prospectively for immunohistochemistry (IHC) experiments 25 ± 4 days post-implantation (Additional file 3: Figure S4f). Figure 4d and e showed that GDCX and not C6 generated higher tumor growth. Validation of tumor size in mouse brain was observed with Hematoxylin and eosin (H&E) staining (Fig. 4e, Additional file 3: Figure S4 g), as cells exhibited classical features of GBM histopathology. GDCX cells notably showed more cells penetrance in the form of greater proportion of extending cells (Fig. 4e). Predictably, the histopathology of GDCX cells in the murine tumor was different from that of the wt-C6 murine glioma tumor; tissues stained with anti-DCX, anti-GFAP, and anti-vimentin antibodies showed a positive correlation with high DCX expression as seen in GDCX tumor (Fig. 4f). DCX-stained brain tumor sections showed higher amount of cells expressing DCX in the nuclear compartment, and these cells are mainly located at the invasive edge of the protruding tumor mass, unlike the wt-C6 tumor where nuclear-DCX positive cells are closer to the center of the tumor mass (Fig. 4g). Furthermore, relatively stronger fluorescence intensity of invasive tumor markers (cd31 and EGFR) was observed in GDCX tumor (Fig. 4h). These in vitro and in vivo functional studies indicate that DCX contributes to the development of invasive gliomas.

### Nuclear import of DCX contributes to glioma development

**Fig. 5:**
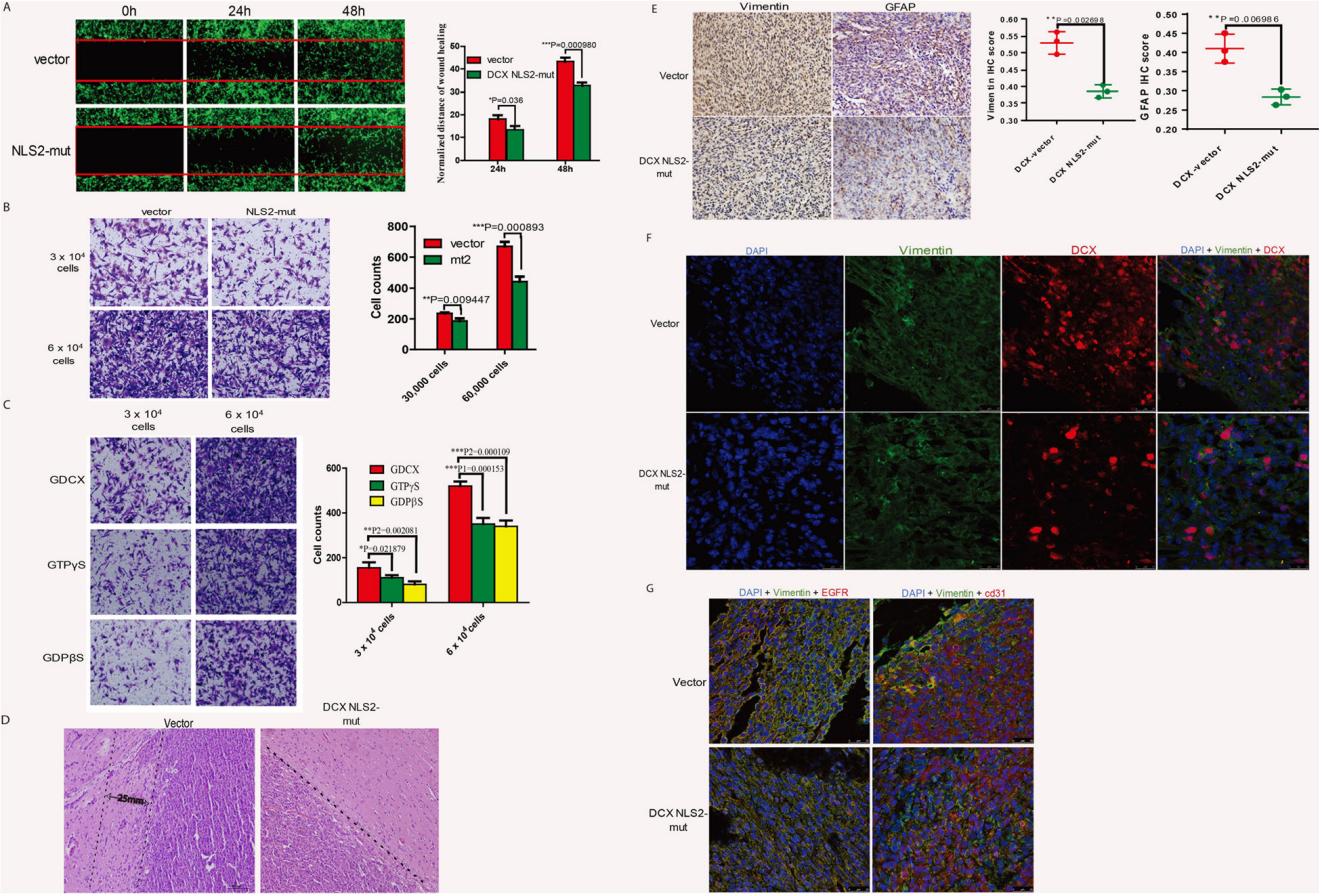
DCX Nuclear Import contributes to GBM development. **a** Wound healing migration assay comparing DCX^+^ U251MG cells with DCX NLS2-mutant cells. t1=3.118, df1=4, t2=8.655, and df2=4. **b** Boyden’s transwell invasion assay involving DCX^+^ vector cells and DCX NLS2-mut cells, quantitated by Image-Pro-Plus. t1=4.680, df1=4, t2=8.868 df2=4. **c** Similar experiments were performed with GDCX-C6 cells treated with GTP analogs. F1=13.508, df1=2, F2=50.392, df2=2. **d** Hematoxylin-Eosin staining of enlarged mice brain section showing infiltrating tumors at the invasive frontiers. The dashed boundary depicts the distance covered by invasive cells. Scale bars, 50μm. **e** Representative immunohistochemical staining to compare GFAP (df=4, t=5.099), or vimentin (df=4, t=6.622) immunoreactivity in vector and DCX NLS2-mut tumors. Scale bars, 25μm. **f** Immunostaining of brain sections showing DCX nuclear accumulation intensity. Scale bars=25μm. **g** Immunofluorescence image of invasive glioma markers (CD31 and EGFR) in DCX-vector and DCX NLS2-mut tumors. Scale bar, 25μm

Since DCX nuclear accumulation only occurs in glioma cells, we investigated the effects of DCX nucleocytoplasmic transport on glioma progression. Wound healing migration assay revealed a retarded migration of DCXNLS2-mut U251 cells compared to DCX-vector cells (Fig. 5a). Furthermore, the number of DCXNLS2-mut cells that invaded the base of the transwell chamber was significantly lower than that of the DCX vector group (Fig. 5b). Transwell experiments replicated with GDCX-C6 cells treated with GTPγS/GDPβS also showed higher invasion of untreated GDCX-C6 cells compared to the treated groups (Fig. 5c). Next, we implanted DCXNLS2-mut cells to be compared with DCX-C6 vector cells in BALB/c nude mice (*n* = 6). Lower luciferase bioluminescence intensity and smaller tumor volume were observed in DCXNLS2-mut mice (Additional file 4: Figure S5a and b). Further observations revealed a relatively smaller tumor cell extension of the tumor mass exhibiting invasive features, while the DCX vector tumor grew diffusely and showed more invasiveness (Fig. 5d). Histopathological examinations on tumor sections showed DCXNLS2-mut tumor tissue displayed less amount of Vimentin and GFAP staining compared with DCX-vector via IHC (Fig. 5e). Even though DCX-NLS2 tumor showed a higher amount of DCX IHC intensity (Additional file 5: Figure S5c), immunofluorescence showed a lesser amount of nuclear DCX in the DCX NLS2-mut tumor, and those cells expressing nuclear DCX were displaced mainly to the core of the tumor body (Fig. 5f). Immunostaining of invasive cell markers showed lower CD31 and EGFR expression in DCX NLS2-mut tumor (Fig. 5 g). Together, these pathological and molecular criteria indicate that nuclear translocation of DCX is pivotal to the pro-oncogenic functions of DCX in gliomas.

### High GDNF expression promotes DCX nuclear import

**Fig. 6:**
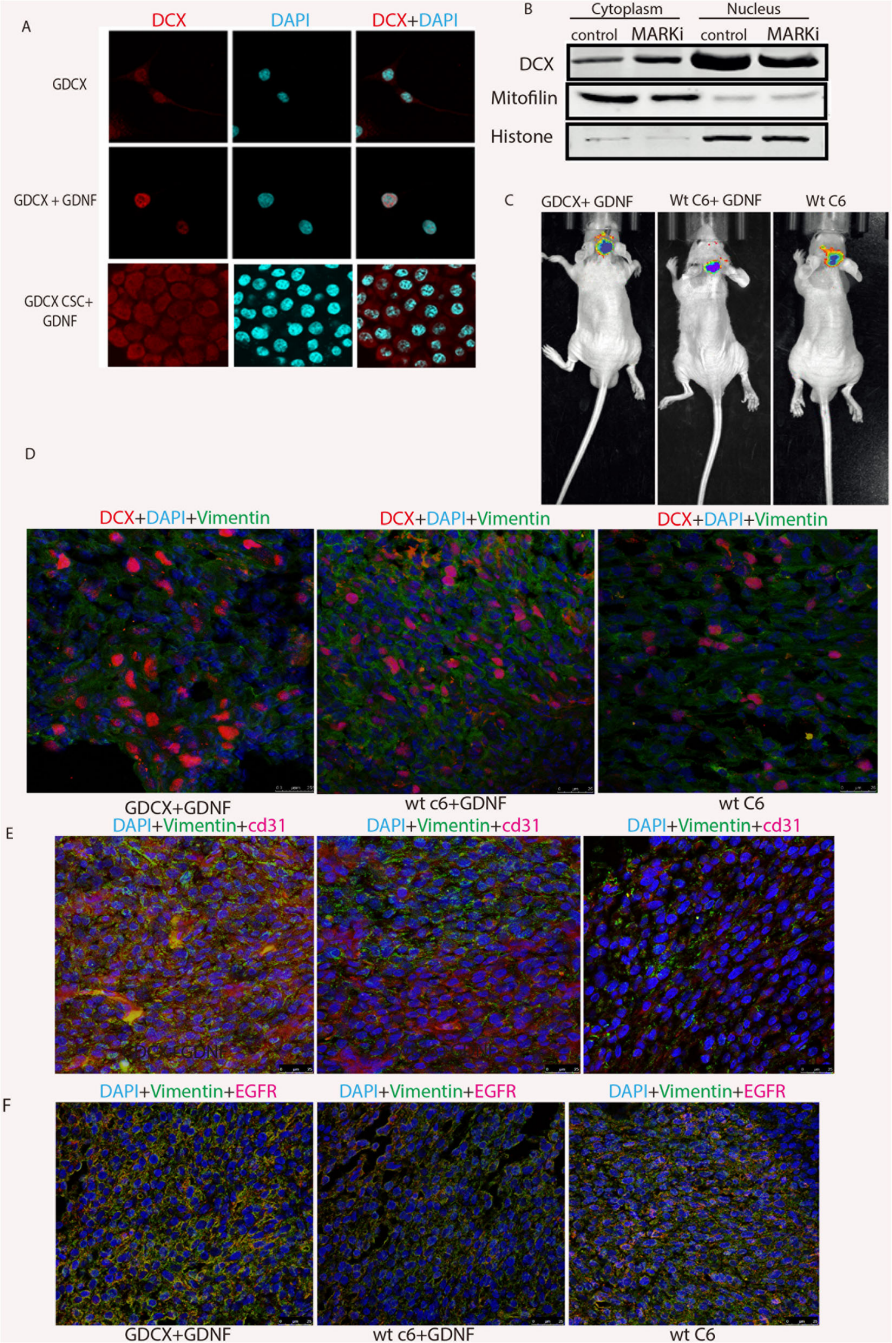
DCX Nuclear import is promoted by GDNF. **a** Immunostaining of GDNF-treated GDCX and GDCXU251 stem cells compared with untreated GDCX cells. Scale bar, 20 μM. **b** Western blotting assay showing DCX nucleocytoplasmic expression in both MARK1-treated and untreated GDCX U251 cells. **c** Representative bioluminescence images comparing GDNF-treated GDCXC6 cells, GDNF-treated-wt C6, and untreated wt C6 cells at week 3 post-implantation. **d** Immunostaining of brain sections showing DCX nuclear accumulation intensity. Scale bars = 25 μm. Immunofluorescence image of invasive glioma markers, CD31 (E), and EGFR (F), Scale bars = 25 μm

Post-translational modifications such as phosphorylation may modulate nuclear import rates, e.g., phosphorylation of adjacent Ser111/112 N-terminal to the NLS of SV40T antigen greatly enhances its nuclear import [41]. We had previously reported that GDNF contributes to glioma progression [2, 31], while GDNF has also been linked to the activation of kinases in favor of glioma [42]. We asked if GDNF can promote DCX phosphorylation and subsequently affect nuclear import. Previous knowledge shows that Ser47 of DCX is phosphorylated by microtubule-affinity regulating kinase-1 (MARK1), and this phosphorylation enhances cellular migration [43]. Exogenous addition of 100 ng/ml recombinant GDNF (Gibco) increased MARK mRNA and protein expression (Additional file 5: Figure S6a and b) and subsequently improved DCX nuclear expression in U251MG cells and stem-like cells derived from GDCX-U251 cells (GDCX CSC) (Fig. 6a). To examine the effects of phosphorylation on DCX nuclear import, GDCX-U251 cells were treated with 1 μM of MARK inhibitor (Carlbiochem) as previously described by Guo et al., [44], the MARK1 inhibition reduced DCX nuclear import (Fig. 6b), and consequently hindered invasive abilities of GDCX-U251 cells (Additional file 5: Figure S6c), however, inhibition of GDNF transcription in U251 cells (by curcumin-induced hypoacetylation of GDNF, as we previously described [45]) still showed nuclear expression of DCX but reduced invasiveness (figure not shown). Then we asked if phosphorylation of DCX on other amino acid residues affects DCX nuclear import. We treated GDCX-U251 cells with SP600125 to inhibit JNK’s phosphorylation of DCX on Thr321, Thr331, Ser332, and Ser334. Immunoblots showed that inhibition of JNK abrogated phosphorylation of DCX on Ser334, blocking GDNF transcription with curcumin only reduced but did not abrogate DCX phosphorylation on ser 334 (Additional file 5: Figure S6d), however, both treatments did not affect DCX nuclear import (Additional file 5: Figure S6e). Similarly, blocking GDNF transcription had no effect on DCX phosphorylation on ser297, while there was little or no effect after GDNF overexpression (Additional file 5: Figure S6f). These observations indicate that GDNF promotes phosphorylation of DCX on Ser47 and aids DCX nuclear import, but is not necessary for DCX nuclear accumulation. This was further validated in rat’s astrocytic cells, wherein the treatment of exogenous GDNF did not induce DCX nuclear translocation. Next, we checked if high GDNF expression promotes DCX activities in vivo. GDCX-C6 cells and wt-C6 cells were cultured in total medium treated with 200 ng/ml of exogenous GDNF before intracranial injection into the BALB/c nude mice (*n* = 12). Further intranasal delivery of recombinant GDNF protein was administered twice a week, starting from day 7 after implantation. In comparison with wt-C6, bioluminescence imaging showed higher tumor intensity at week 3 post-injection (Fig. 6c). Tissue immunoblots showed higher DCX nuclear accumulation in high GDNF groups than in untreated C6 xenografts (Fig. 6d), as well as higher invasiveness marker (cd31) (Fig. 6e). EGFR expression in GDNF-treated groups was also higher than in the untreated group (Fig. 6f). These data show that high GDNF expression aids nuclear import of DCX in glioma cells.

## Discussion

Since the first report on DCX expression in gliomas, its functions in glioma development have remained exclusive. Based on the conflicting reports from Santra et al., and that of Xu and colleagues that established their research on the former’s reports [14–18, 32], we verified the expression of DCX in gliomas for proper clarifications. Our results and analysis from the Oncomine database corroborate reports from Rich et al., [10–13] and others as we report here that DCX is expressed in glioma tissues and conventional cell lines.

We have also identified here that DCX shuttles to the nucleus of glioma cells, and it requires specific residues between ser^**47**^ and tyr^**70**^ in a RanGTPase dependent manner. This is documented here not only in our in vitro binding studies but also by the subcellular fractionation experiments using dcas9-transfected cells where overexpression of DCX resulted in increased migration and invasion of glioma cells in vitro and tumor proliferation in vivo. These results identify a mechanism by which DCX can be transported from the cytoplasm through the NPC to the nucleus subsequent to its association with importin and Ran. It is well established that the role of Ran in nucleocytoplasmic transport of molecules is to regulate the cargo binding to- and dissociation from- the transport complex [46]. In this study, DCX was capable of partially binding to Ran without the aid of GTP or GDP, which may explain the stability of the import. Moreover, when GDP phosphorylation is blocked, importin-β still binds to DCX. This is proven by the co-IP experiments shown in Fig. 2. This may mean that the interactive point of DCX-importin-α and DCX-importin-β are partially redundant. Another explanation to this is that DCX-importin-β binding occurs in the nucleus for the activation of DCX dissociation from the complex or possible return of DCX to the cytoplasm.

From the two putative DCXNLSs, DCXNLS2 was confirmed to be a functional DCXNLS. This NLS sequence is comprehensible because of its structural position in the context of full-length DCX, and its resemblance to the classical SV40 T antigen NLS [47, 48]. Hence, we concluded that DCX requires a cNLS for its nuclear translocation. Another method of validating a putative cNLS is the direct binding of cargo to the import receptors via the NLS. DCXNLS2 facilitated the binding of DCX to importin-α, as proven by the mutant DCXNLS2 failure to bind with the import receptors; altering DCXNLS2 consequently restricted binding of DCX to the nuclear import receptors (Fig. 3), and this subsequently inhibited the progression of DCX nuclear import. The interaction of DCX to importin was further illustrated with the *in-silico* protein-protein docking model presented in Additional file 3: Figure S4 confirming DCX-importin interactions on DCX NLS2 residues. This DCX NLS2 extension (Ser47-Tyr70) suggests that the DCX NLS is a bipartite cNLS. The predicted DCXNLS1, on the other hand, is not a DCX NLS; DCX interactions with nuclear transport molecules remained intact when this putative sequence was mutated or deleted. Also, mutation or deletion of specific residues on the DCXNLS2 region had a significant negative influence on the DCX protein expression in vivo; hence, it is safe to assume that a portion of the expressed DCX is endogenous wt-DCX which can be observed in the nucleus of few cells. Whether DCX possesses an NES or shuttles the nuclear pore complex (NPC) is still unknown. Hence, further studies on the detailed interactions of DCX with nuclear proteins are imperative.

Cellular behaviors are controlled by cues acting as growth-promoting or antagonists and growth preventing/repellents actively regulating or deregulating free-flowing kinases in gliomas [49]. Our biochemical assays herein raise essential concepts related to the role of these cues and how glioma cells may interpret and respond to them. Systematically, it appears that DCX trafficking may be accelerated by phosphorylation at DCX ^**ser47**^ by MARK. The high expression of GDNF and the free flow of MARK1 may enable the phosphorylation of DCX on ser47 before the trafficking of DCX into the nucleus. DCX is generally regarded as a cytoplasmic protein, at least until now. Specific conditions such as elevated MARK expression or chemokine stimulation may be expedient in DCX translocation.

Having established the detailed processes involved in the nuclear transport of DCX, its cellular functions in the nucleus becomes an open question. Generally, pro-oncogenic proteins that can translocate from the cytoplasm to the nucleus are usually involved in distinct cellular functions in these subcellular compartments [1]. DCX translocation improves cellular invasion and proliferation in vitro and in vivo, as overexpression of DCX and inhibition of its translocation to the nucleus respectively promotes or hinders migration and invasion of glioma cells. Furthermore, DCX expression alone may be considered as a poor prognostic marker of GBM, inhibiting DCX nuclear accumulation may weaken the invasive abilities of glioma cells and pave the way for effective delivery of therapeutic interventions. In summary, our data pointed out a previously unknown DCX role and localization pattern in GBM biology, which plays a central role in migration, invasion, and proliferation. It is predictable that research on DCX and its pro-oncogenic functions will be further appreciated with a broad interest in its research with regards to glioma development.

## Conclusions

In the present study, we further provide evidence on the expression of DCX in glioma cells and report a nexus between DCX expression and glioma development. For the first time, we report that DCX is expressed in the nucleus of glioma cells via the RanGTPase signaling, consequently promoting glioma progression. In accordance with our discovery that DCX expression in the nucleus is via a cytoplasm-nucleus movement, we identified a novel NLS required by DCX for its nucleocytoplasmic movement in glioma cells amongst other possible NLS as we also characterize point of interactions on the DCX NLS with import receptors in the buildup to the nuclear import of DCX (Ser47-Tyr70). This study also reveals an additional role for GDNF in GBM progression. GDNF can promote DCX nuclear transport by activating MARK’s phosphorylation of ser47 residue flanking the DCX NLS. These findings demonstrate that DCX is pro-oncogenic and can serve as a future therapeutic target in gliomas as we describe a consistent observation between inhibition of DCX nucleocytoplasmic transport and a decline in the GBM cell population.

Based on the extensive study of DCX in neuronal development and its crucial roles in adult neurogenesis, this study opens a broader interest in possible exploitation of DCX functions in neurons by glioma communication systems.

## Supplementary information

**Additional file 1: Figure S1.** Datasets from Oncomine cancer profiling database comparing mRNA levels (log2 median-centered intensity) of human DCX expression showing: Beroukhim brain statistics in the normal brain vs DCX expression in glioblastoma tissues, t = 1.813, fc = 1.089, or normal brain tissue vs anaplastic astrocytoma, t = 3.031, fc = 1.297; Shai brain statistics in the white matter vs glioblastoma tissues (GBM), t = 4.066, fc = 2.349, or white matter vs Astrocytoma, t = 2.926, fc = 1.983; and Liang brain statistics in the brain vs glioblastoma, t = 3.160, fc = 3.232.

**Additional file 2: Figure S3.** Predicted nuclear localization sequences from NLStradamus (A), seqNLS (B), and Loctree (C) databases. (D) DCX structure and function as predicted by the PredictProtein database. (E) Ribbon and stick computer-simulated human DCX N-terminal structure (1MJD.pdb) visualized by PyMOL Molecular Graphics System. DCX-importin-α Interactions based on DCX and importin subfamily’s 3D structure docking on HDOCK server. Ribbon and stick representation of DCX (green) and importin-α1 (jade) (F), importin-α3 (G), importin-α5 (H), importin-α6 (I), and importin-α7 (J). Figsures F-J shows the interacting residue between DCX and importin-α in surface format, the surface format showing the interacting residues, as well as illustration of the electrostatic interaction (red represents negatively charged area and blue represents positively charged area).

**Additional file 3: Figure S4.** (A) Invasion assay conducted with CD133^+^ stem cells generated from dcas9 induced DCX-overexpression C6 cells (GDCX-GBSC), and GBSC, stem cells generated from wt C6 cells using 8 μm pore Boyden transwell chamber and the quantification of invasive cells at the lower chamber (t = 35.439, df = 4). (B) Differential neurospheres formation abilities of DCX^+^ cells vs. wt C6 cells. Neurospheres were counted day 5 following induction into stem-like cells. Scale bars = 100 μm. day4 (t = 7.140, df = 4), day 5 (t = 8.902, df = 4). (C) CCK-8 viability test for cell proliferation was conducted in GDCX-C6 cells compared with wt-C6 cells (t = 4.999, df = 4), and GBSC vs GDCX SC at day4 (t = 8.164, df = 4), and day 5 (t = 7.152, df = 4). (D) Cell viability test conducted in U251 DCX-siRNA cells vs wt-U251 (t1 = 17.602, t2 = 16.358, df = 5) (E) Cell viability conducted with untreated U251 DCX-siRNA cells compared with DCX-treated cells (t = 20.903, df = 5) (F) Images of BALB/c nude mice before sacrifice (G) Comparison between tumor sizes in GDCX xenograft and wt-C6 xenograft (t = 6.598, df = 4).

**Additional file 4: Figure S5.** (A) Comparisons between representative bioluminescence images of BALB/c nude mice injected with either DCX vector or DCXNLS2-mutant cells. (B) Hematoxylin-Eosin staining of 8 μm tissue slices of mice brain and comparison between tumor sizes (t = 17.47, df = 4). (C) Immunohistochemical staining to compare DCX immunoreactivity in vector and DCX NLS2-mut tumors. Scale bars, 25 μm. (df = 4, t = 4.238).

**Additional file 5: Figure S6.** (A) mRNA expression of MARK1 in GDNF-treated GDCXU251 cells. 6 h treatment was chosen for subsequent experiments. (B) Immunoblots analysis showing protein expression of MARK1 in GDNF-treated and untreated GDCX cells, t = 6.461, df = 5. (C). Boyden’s invasion assay and the quantitation of invasive untreated GDCX and MARK inhibitor-treated GDCX cells (t = 19.66, df = 4). (D) Immunoblots showing phosphorylated DCX at serine 334 (pDCX ser334) in GDNF-treated GDCX cells, untreated, curcumin-treated, and JNK inhibitor-treated cells. (E) Subcellular localization of DCX in JNK inhibitor-treated-, curcumin treated-, or untreated-GDCX cells as detected by immunoblotting. (F) Immunoblots showing phosphorylated DCX at serine 297 (pDCX ser297) in GDNF-treated or untreated-GDCX cells, and curcumin-treated GDCX cells.

**Additional file 6: Table S1.** Comparison of the sequence of recombinant expression vectors of DCX wild type and DCX variant lentiviruses.

## Data Availability

The supplementary table and Figures are attached.

## Abbreviations

CCK8: Cell Counting Kit-8
CD31: Platelet endothelial cell adhesion molecule (PECAM-1) also known as cluster of differentiation 31
cNLS: Classical Nuclear localization sequence/signal
CRISPR: Clustered Regularly Interspaced Short Palindromic Repeats
EGFR: Epidermal growth factor receptor
GFAP: Glial fibrillary acidic protein
GTPγS: γ-glutamyltransferase
HA-h: Human Astrocytes-hippocampal
IHC: Immunohistochemistry
NLS-del: Nuclear Localization Sequence- deletion
NLS-mut: Nuclear Localization Sequence-mutant
PulSA: Pulse Shape Analysis
RA: Rat’s astrocytes
ser: Serine
tyr: GDPβS: Tyrosine; 5′- [β -thio] diphosphate trilithium salt
