# Supplementary figures and images for: Doublecortin undergo nucleocytoplasmic transport via the RanGTPase signaling to promote glioma progression
Source: Cell Commun Signal. 2020 Feb 12;18:24. doi: 10.1186/s12964-019-0485-5 (PMC7017634; doi:10.1186/s12964-019-0485-5)

Supplementary Figure 1

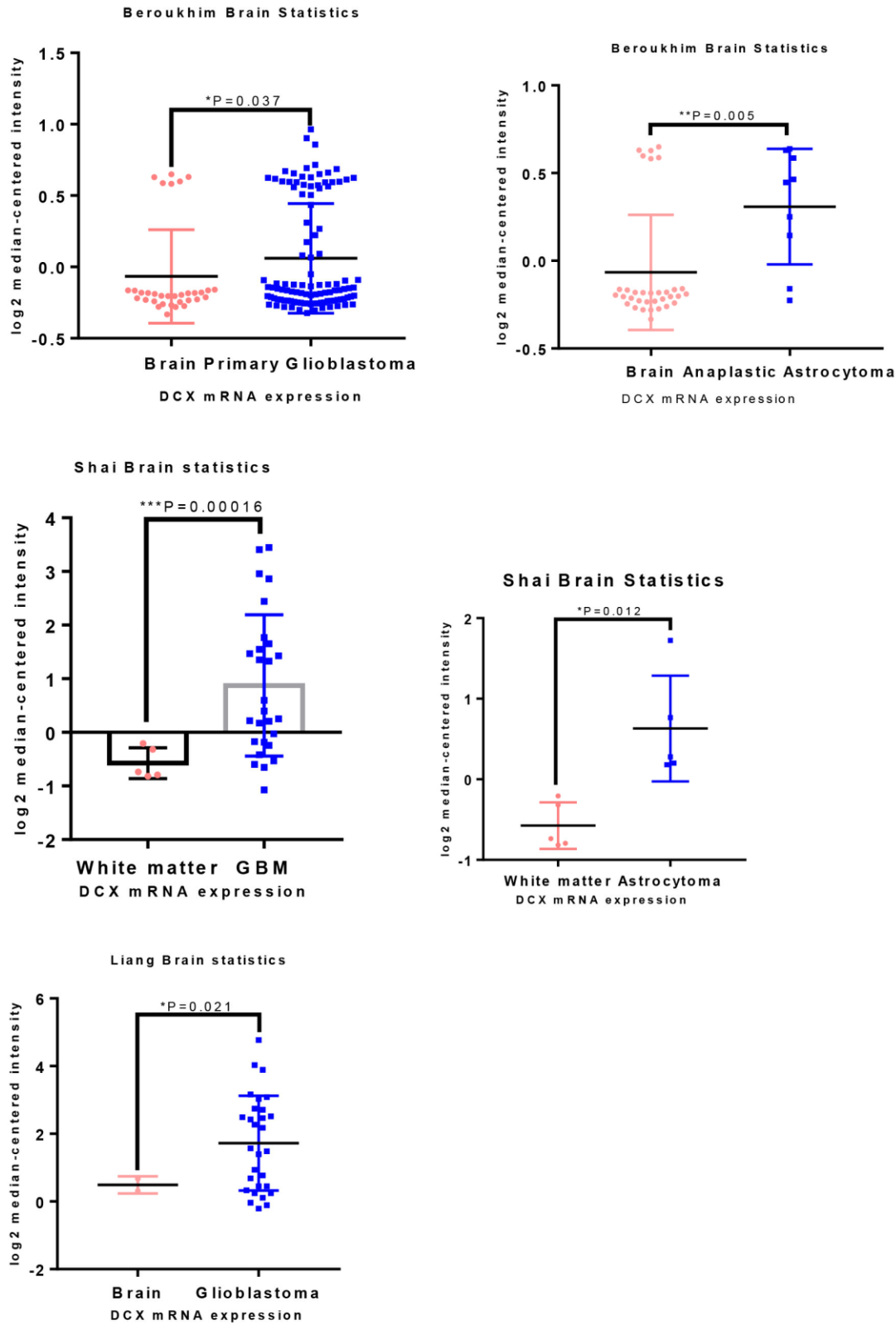

Supplement: Supplementary file 2 — Additional file 1: Figure S1. Datasets from Oncomine cancer profiling database comparing mRNA levels (log2 median-centered intensity) of human DCX expression showing: Beroukhim brain statistics in the normal brain vs DCX expression in glioblastoma tissues, t = 1.813, fc = 1.089, or normal brain tissue vs anaplastic astrocytoma, t = 3.031, fc = 1.297; Shai brain statistics in the white matter vs glioblastoma tissues (GBM), t = 4.066, fc = 2.349, or white matter vs Astrocytoma, t = 2.926, fc = 1.983; and Liang brain statistics in the brain vs glioblastoma, t = 3.160, fc = 3.232. [file 12964_2019_485_MOESM2_ESM.pdf]

A

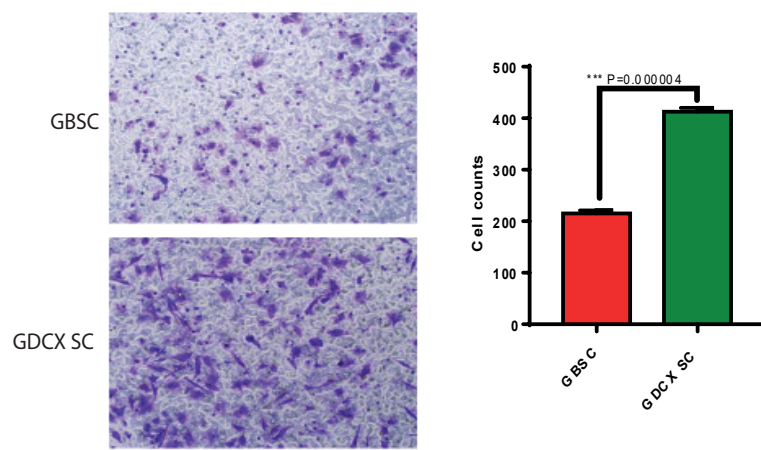

B

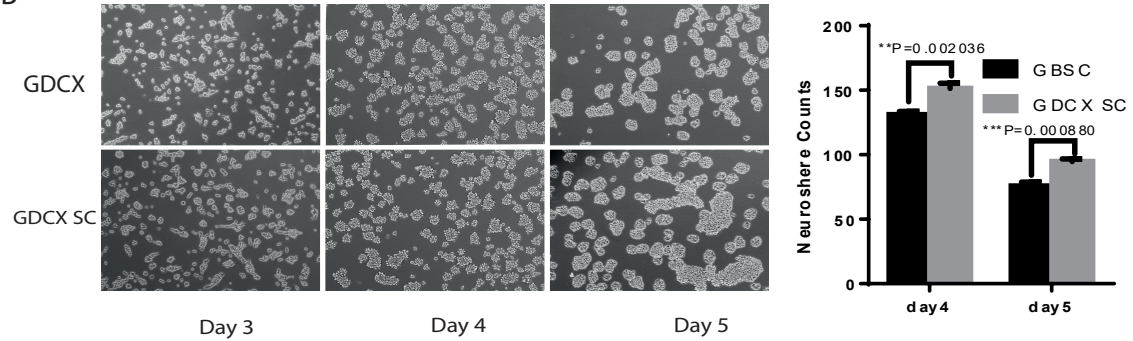

C

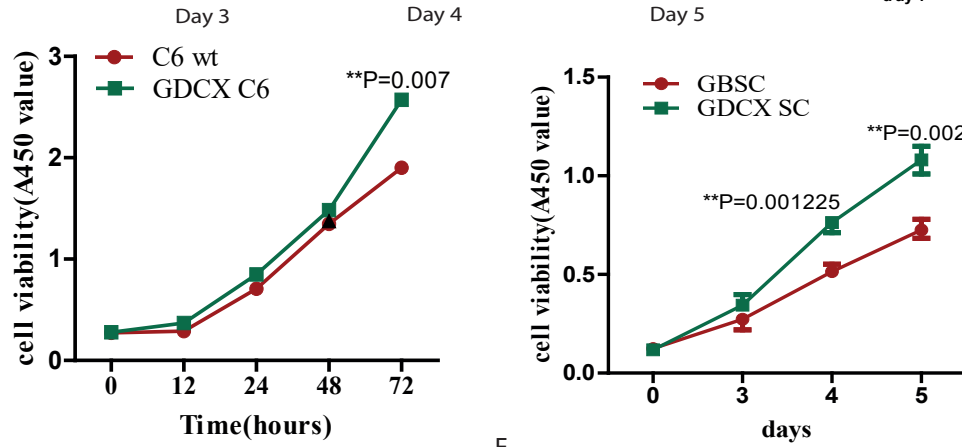

D

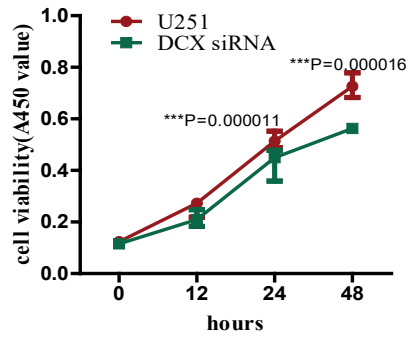

E

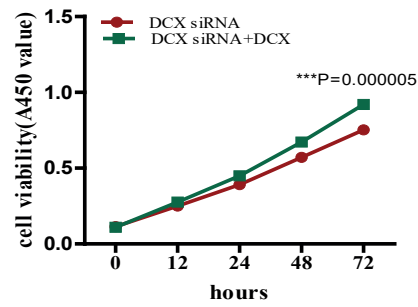

F

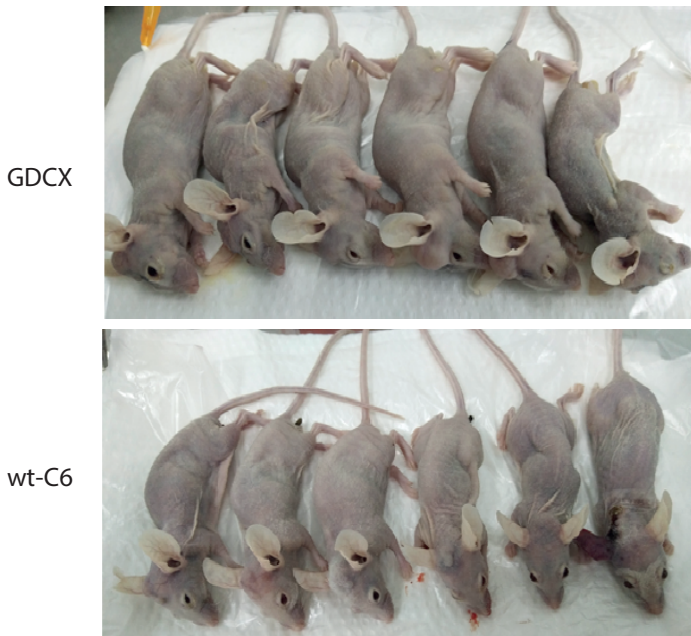

G

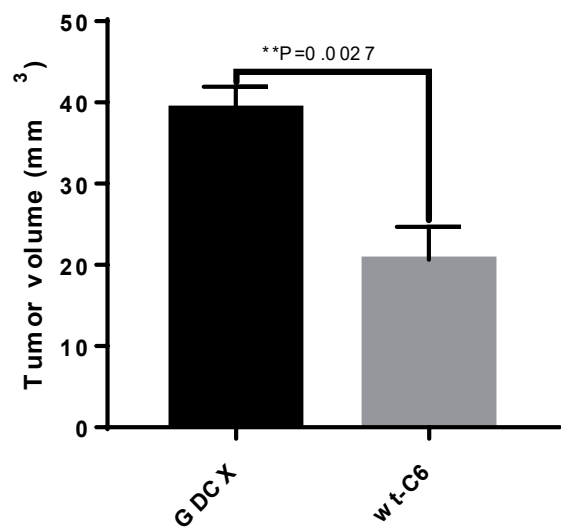

Supplement: Supplementary file 4 — Additional file 3: Figure S4. (A) Invasion assay conducted with CD133+ stem cells generated from dcas9 induced DCX-overexpression C6 cells (GDCX-GBSC), and GBSC, stem cells generated from wt C6 cells using 8 μm pore Boyden transwell chamber and the quantification of invasive cells at the lower chamber (t = 35.439, df = 4). (B) Differential neurospheres formation abilities of DCX+ cells vs. wt C6 cells. Neurospheres were counted day 5 following induction into stem-like cells. Scale bars = 100 μm. day4 (t = 7.140, df = 4), day 5 (t = 8.902, df = 4). (C) CCK-8 viability test for cell proliferation was conducted in GDCX-C6 cells compared with wt-C6 cells (t = 4.999, df = 4), and GBSC vs GDCX SC at day4 (t = 8.164, df = 4), and day 5 (t = 7.152, df = 4). (D) Cell viability test conducted in U251 DCX-siRNA cells vs wt-U251 (t1 = 17.602, t2 = 16.358, df = 5) (E) Cell viability conducted with untreated U251 DCX-siRNA cells compared with DCX-treated cells (t = 20.903, df = 5) (F) Images of BALB/c nude mice before sacrifice (G) Comparison between tumor sizes in GDCX xenograft and wt-C6 xenograft (t = 6.598, df = 4). [file 12964_2019_485_MOESM4_ESM.pdf]

A

Vector

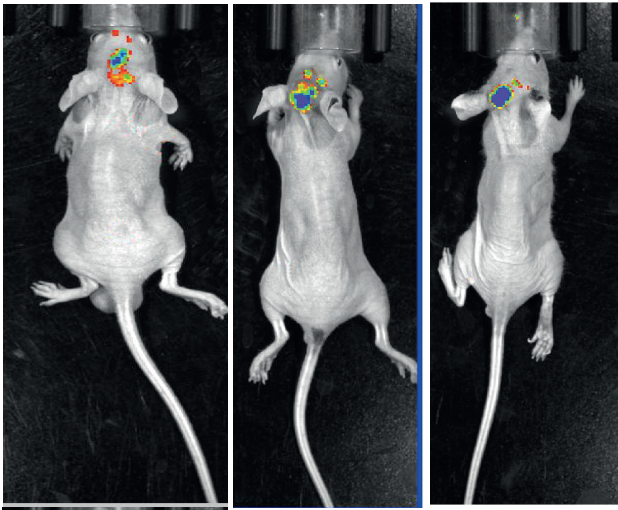DCX NLS2-  
mut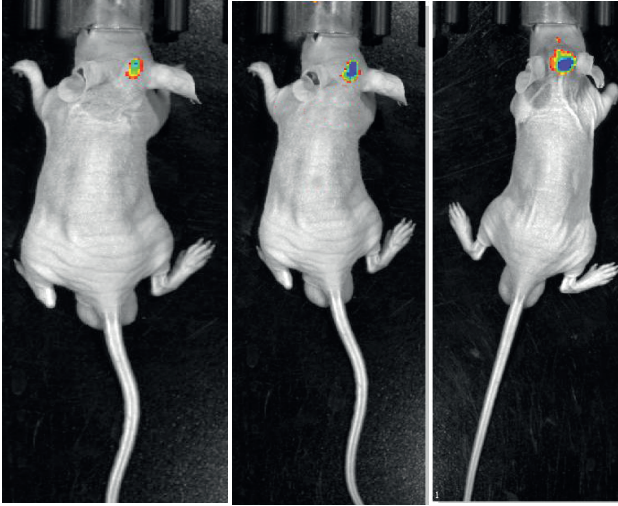

B

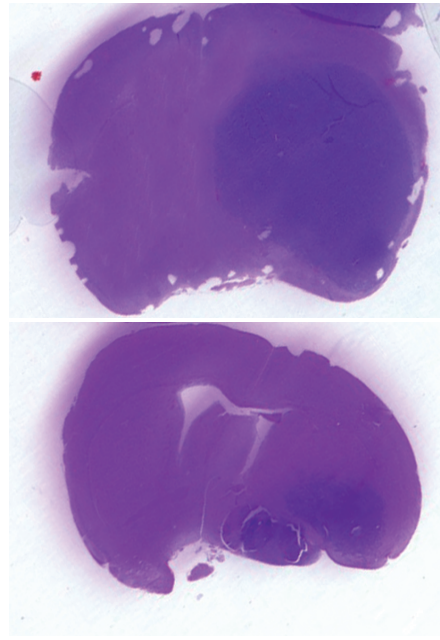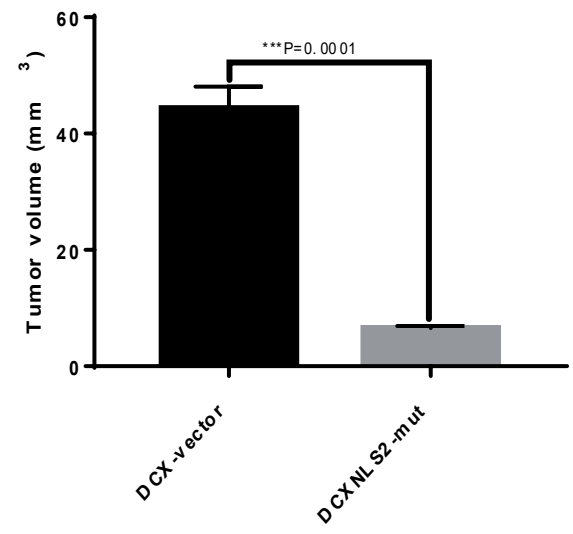

C

Vector

DCXNLS2-mut

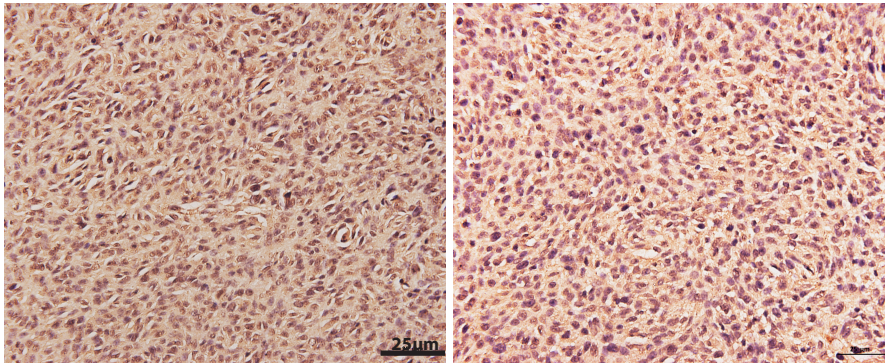

DCX

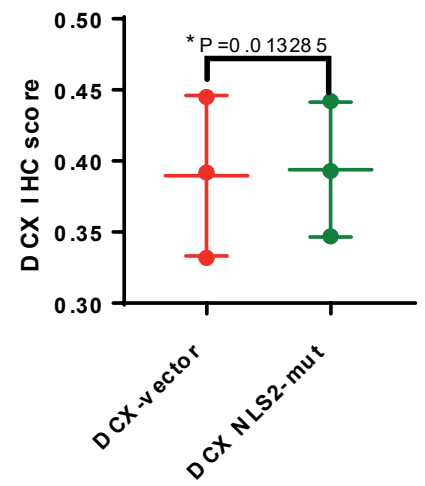

Supplement: Supplementary file 5 — Additional file 4: Figure S5. (A) Comparisons between representative bioluminescence images of BALB/c nude mice injected with either DCX vector or DCXNLS2-mutant cells. (B) Hematoxylin-Eosin staining of 8 μm tissue slices of mice brain and comparison between tumor sizes (t = 17.47, df = 4). (C) Immunohistochemical staining to compare DCX immunoreactivity in vector and DCX NLS2-mut tumors. Scale bars, 25 μm. (df = 4, t = 4.238). [file 12964_2019_485_MOESM5_ESM.pdf]

Supplementary Figure 6

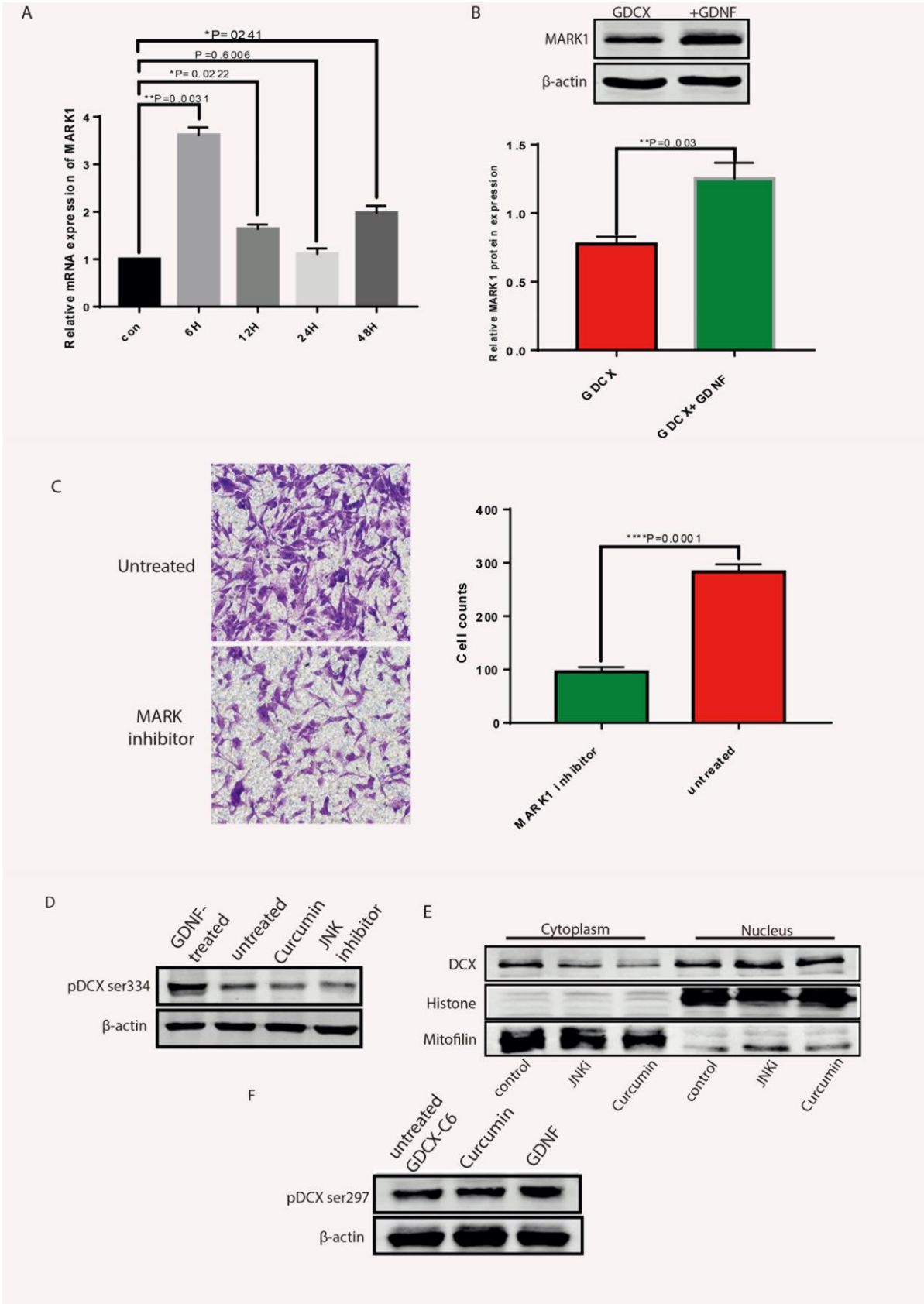

Supplement: Supplementary file 6 — Additional file 5: Figure S6. (A) mRNA expression of MARK1 in GDNF-treated GDCXU251 cells. 6 h treatment was chosen for subsequent experiments. (B) Immunoblots analysis showing protein expression of MARK1 in GDNF-treated and untreated GDCX cells, t = 6.461, df = 5. (C). Boyden’s invasion assay and the quantitation of invasive untreated GDCX and MARK inhibitor-treated GDCX cells (t = 19.66, df = 4). (D) Immunoblots showing phosphorylated DCX at serine 334 (pDCX ser334) in GDNF-treated GDCX cells, untreated, curcumin-treated, and JNK inhibitor-treated cells. (E) Subcellular localization of DCX in JNK inhibitor-treated-, curcumin treated-, or untreated-GDCX cells as detected by immunoblotting. (F) Immunoblots showing phosphorylated DCX at serine 297 (pDCX ser297) in GDNF-treated or untreated-GDCX cells, and curcumin-treated GDCX cells. [file 12964_2019_485_MOESM6_ESM.pdf]
