# Supplementary material for: Doublecortin undergo nucleocytoplasmic transport via the RanGTPase signaling to promote glioma progression
Source: Cell Commun Signal. 2020 Feb 12;18:24. doi: 10.1186/s12964-019-0485-5 (PMC7017634; doi:10.1186/s12964-019-0485-5)
Supplement: Supplementary file 3 — Additional file 2: Figure S3. Predicted nuclear localization sequences from NLStradamus (A), seqNLS (B), and Loctree (C) databases. (D) DCX structure and function as predicted by the PredictProtein database. (E) Ribbon and stick computer-simulated human DCX N-terminal structure (1MJD.pdb) visualized by PyMOL Molecular Graphics System. DCX-importin-α Interactions based on DCX and importin subfamily’s 3D structure docking on HDOCK server. Ribbon and stick representation of DCX (green) and importin-α1 (jade) (F), importin-α3 (G), importin-α5 (H), importin-α6 (I), and importin-α7 (J). Figsures F-J shows the interacting residue between DCX and importin-α in surface format, the surface format showing the interacting residues, as well as illustration of the electrostatic interaction (red represents negatively charged area and blue represents positively charged area). [file 12964_2019_485_MOESM3_ESM.pdf]

A

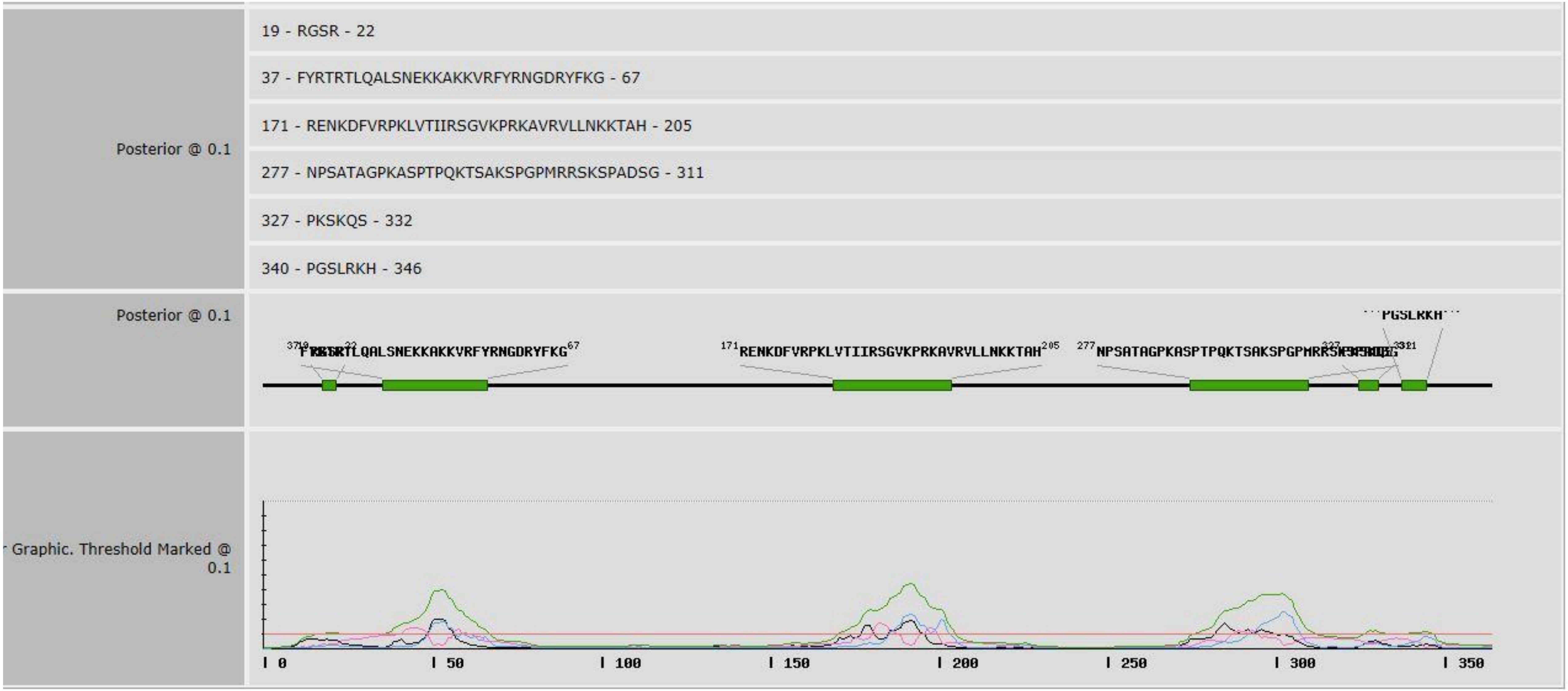
Posterior @ 0.1
19 - RGSR - 22
37 - FYRTRTLQALSNEKKAKKVRFYRNGDRYFKG - 67
171 - RENKDFVRPKLVTIIRSGVKPRKAVRVLLNKKTAH - 205
277 - NPSATAGPKASPTPQKTSAKSPGPMRRSKSPADSG - 311
327 - PKSKQS - 332
340 - PGSLRKH - 346
Posterior @ 0.1
PGSLRKH
FYRTRTLQALSNEKKAKKVRFYRNGDRYFKG
RENKDFVRPKLVTIIRSGVKPRKAVRVLLNKKTAH
NPSATAGPKASPTPQKTSAKSPGPMRRSKSPADSG
Graphic. Threshold Marked @ 0.1
0
50
100
150
200
250
300
350

B

Prediction result (The predicted NLS(s) are underlined)

NP_445831.3+neuronal+migration+protein+doublecortin+[Rattus+norvegicus]: MELDFGHFDERDKASRNMRGSRMNGLPSPTHSAHCSFYRTRTLQALSNEKKAKKVRFYRNGDRYFKGIVY

Definition of different colors in predictions

score range	color
0.1-0.3	
0.3-0.5	
0.5-0.7	
0.7-0.8	
0.8-0.86	
0.86-0.89	
>0.89	

The predicted NLS(s) (score cutoff = 0.8)

Protein ID	Predicted NLS	Start	Stop	The highest score of matches within the prediction
NP_445831.3+neuronal+migration+protein+doublecortin+[Rattus+norvegicus]	GSRMNG	20	25	0.888
NP_445831.3+neuronal+migration+protein+doublecortin+[Rattus+norvegicus]	SNEKKAKKVR	47	56	0.849

C

LOCTREE 3

Protein Subcellular Localization Prediction System

Protein ID	Score	Expected Accuracy	Localization Class	Gene Ontology Terms	Annotation Type
NP_445831.3	67	94%	cytoplasm	cytoplasm GO:0005737(IEA); cytoskeleton GO:0005856(IEA);	PSI-BLAST

Predicted Localization: cytoplasm

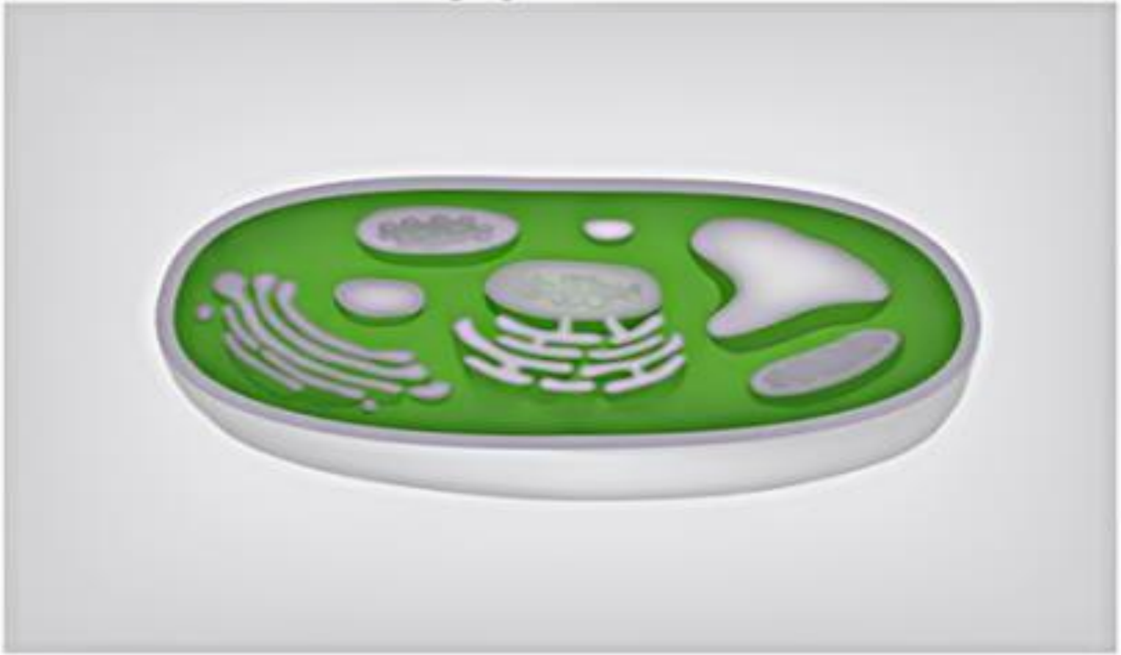

```
 Identities = 245/362 (67%), Positives = 287/362 (79%), Gaps = 29/362 (8%)

Query: 3    LDFGHFDERDKASR--NMRGS------------RMNGL-PSPTHSAHCSFYRTRTLQALS 47
             ++  HF+ERDK  R  + RG+            + NGL PSP HSAHCSFYRTRTLQALS
Sbjct: 7    IELEHFEERDKRPRPGSRRGAPSSSGGSSSSGPKGNGLIPSPAHSAHCSFYRTRTLQALS 66

Query: 48   NEKKAKKVRFYRNGDRYFKGIVYAVSSDRFRSFDALLADLTRSLSDNINLPQGVRYIYTI 107
             +EKKAKK RFYRNGDRYFKG+V+A+SSDRFRSFDALL +LTRSLSDN+NLPQGVR IYTI
Sbjct: 67   SEKKAKKARFYRNGDRYFKGLVFAISSDRFRSFDALLIELTRSLSDNVNLPQGVRTIYTI 126
```

D

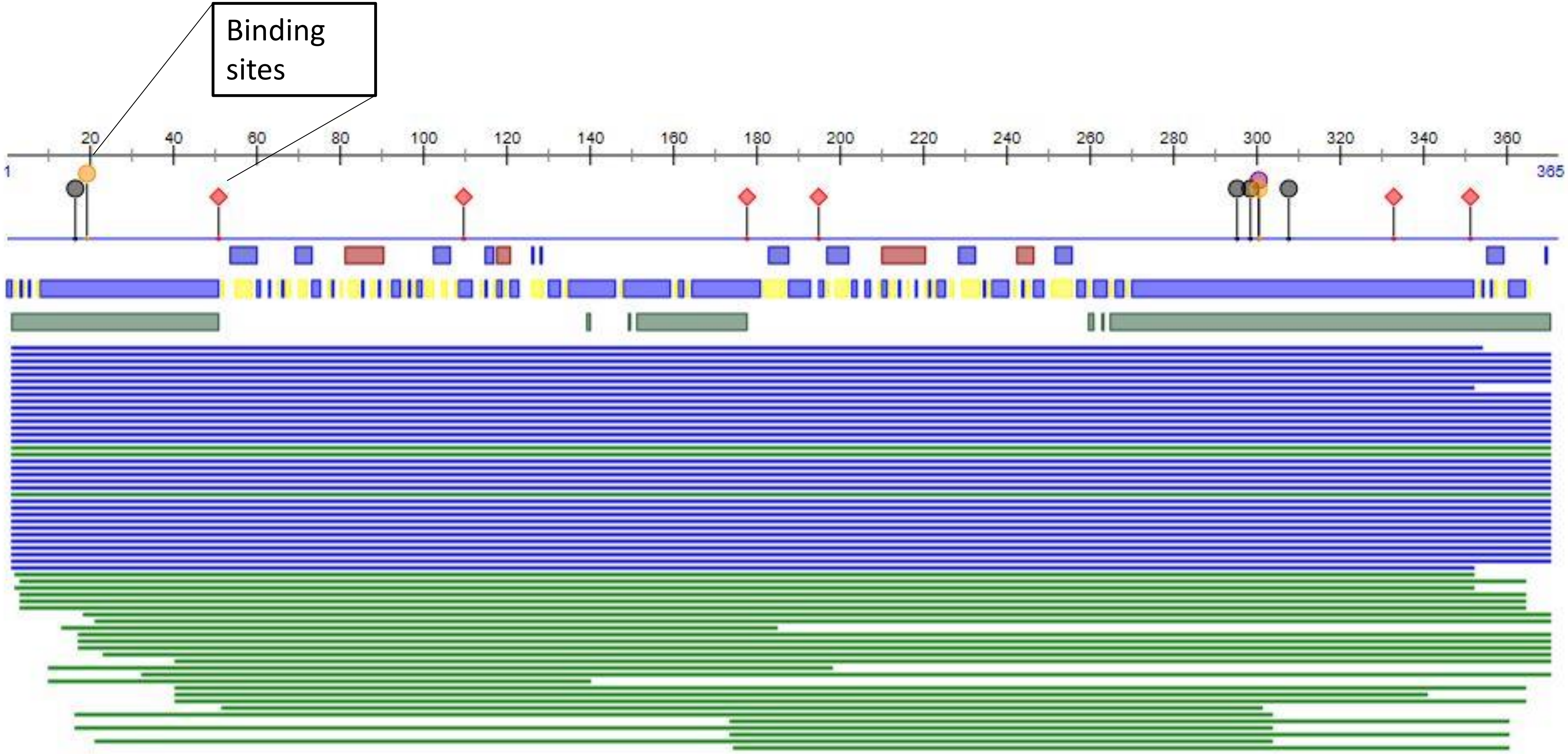
Binding sites
20
40
60
80
100
120
140
160
180
200
220
240
260
280
300
320
340
360
1
365

E

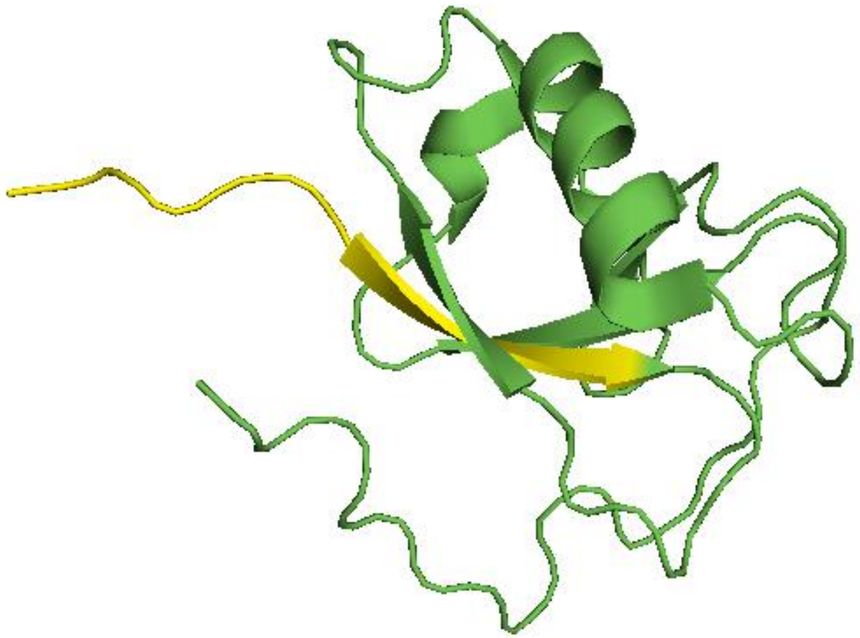

F

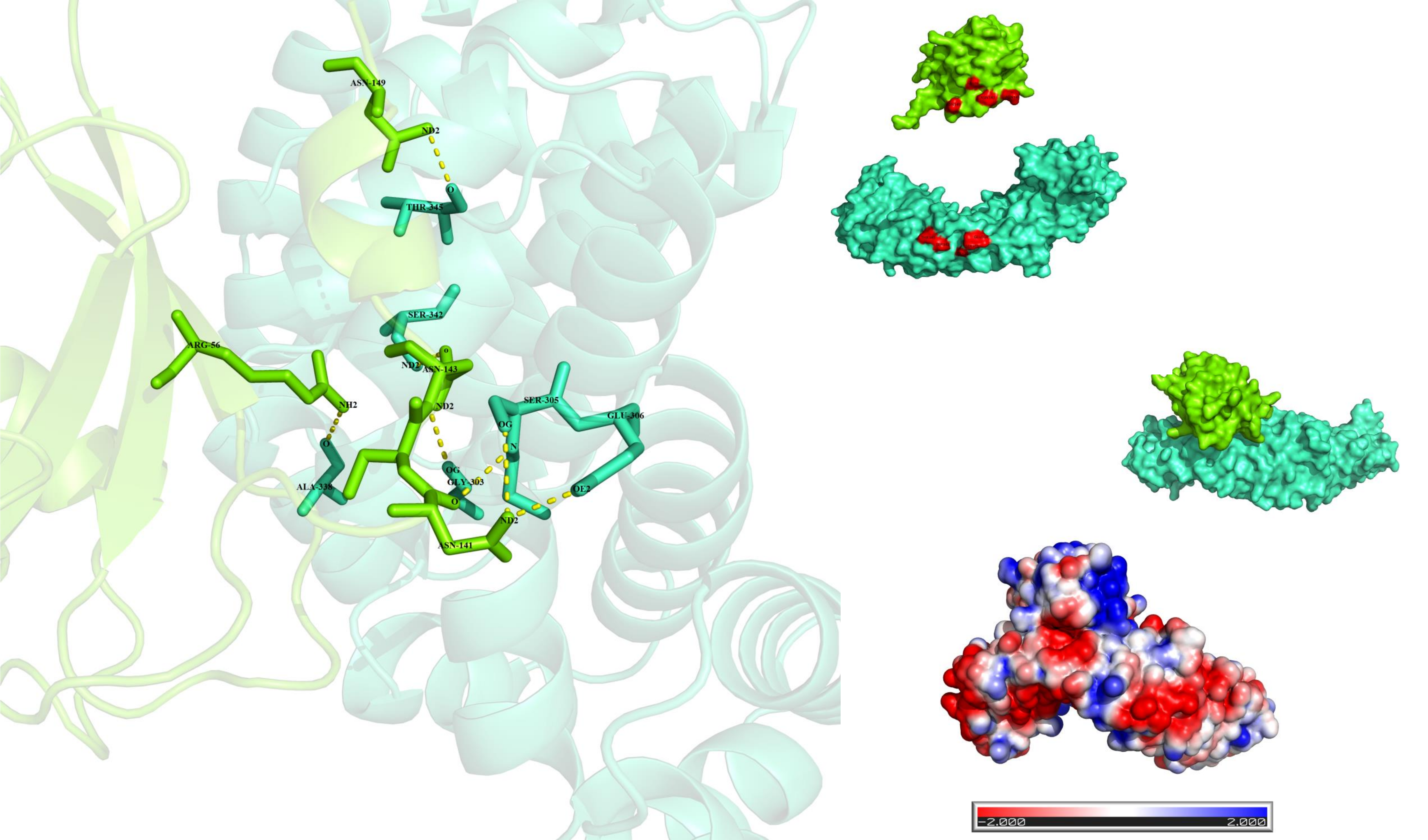
ASN-149
ND2
O
THR-345
SER-342
ARG-56
O
ND2
ASN-143
NH2
ND2
SER-305
GLU-306
OG
O
N
OG
GLY-303
ALA-338
O
OE2
ND2
ASN-141
-2.000
2.000

G

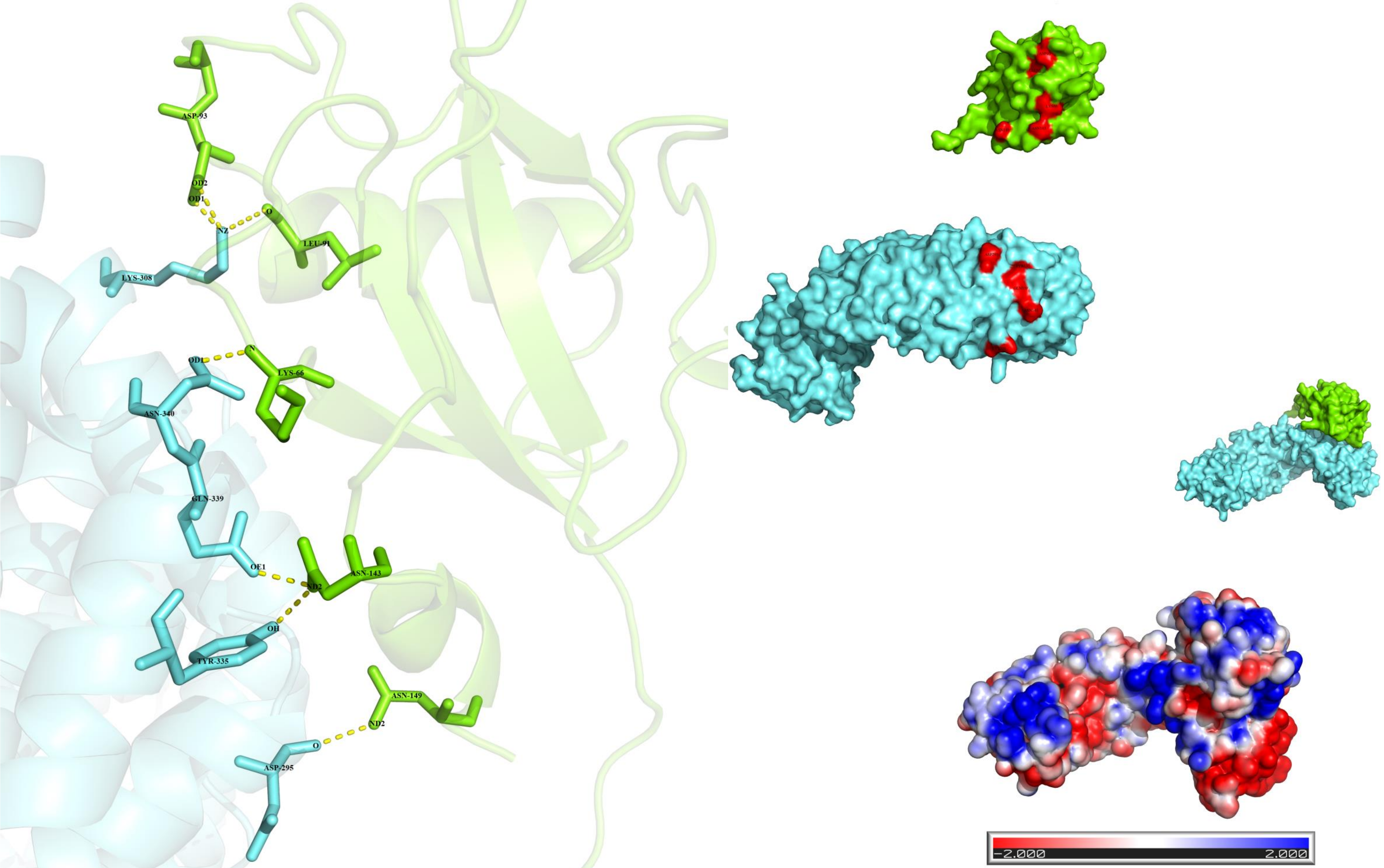

ASP-93
OD2
OD1
NZ
O
LEU-91
LYS-308
OD1
N
LYS-66
ASN-340
GLN-339
OE1
ND2
ASN-143
OH
TYR-335
ASN-149
ND2
O
ASP-295
-2.000
2.000

H

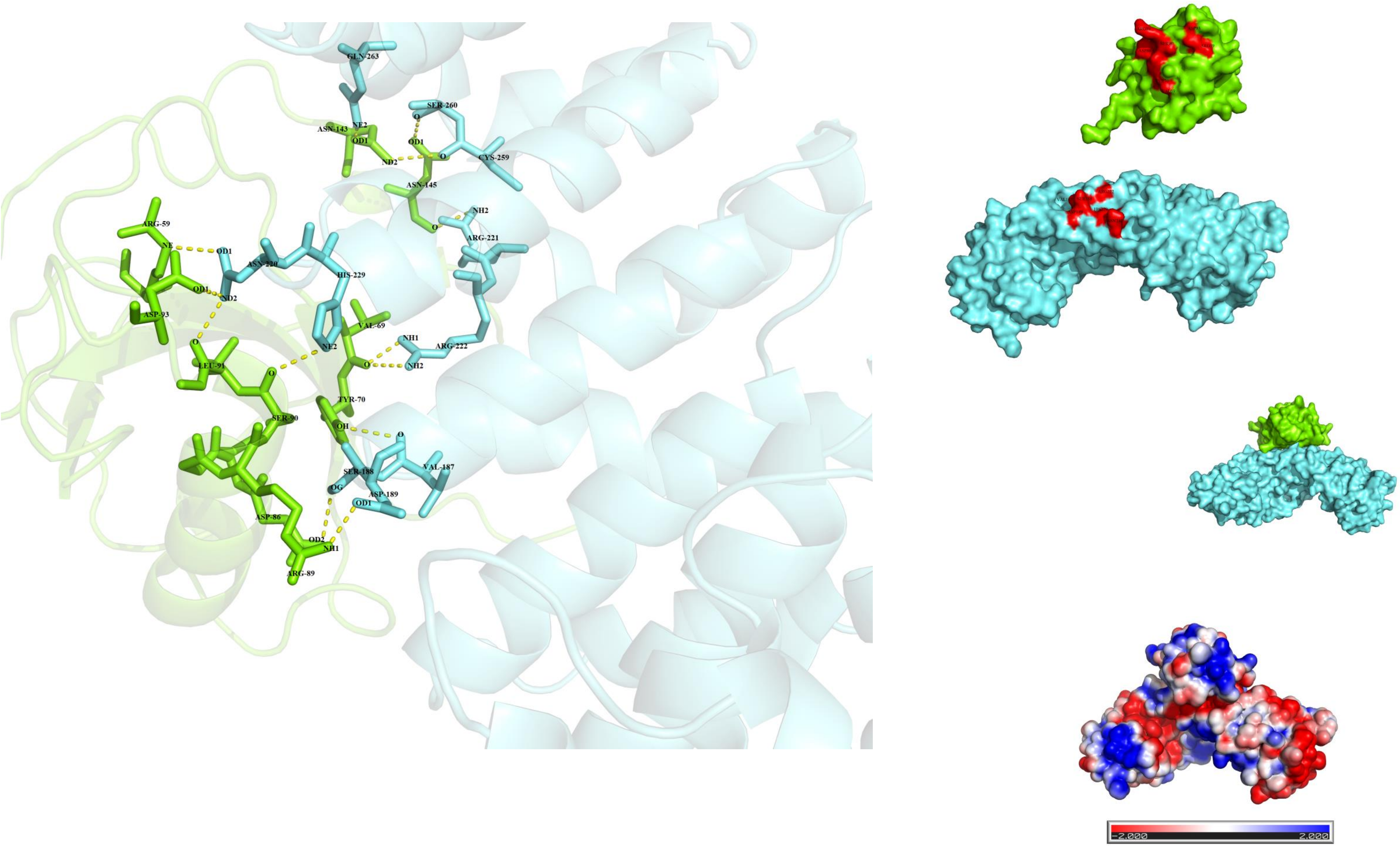

GLN-263
SER-260
ASN-143
NE2
OD1
OD1
ND2
O
CYS-259
ASN-145
NH2
O
ARG-221
ARG-59
NE
OD1
ASN-220
HIS-229
OD1
ND2
ASP-93
VAL-69
O
NH1
ARG-222
NE2
LEU-91
O
O
NH2
TYR-70
SER-90
OH
O
VAL-187
SER-188
OG
ASP-189
OD1
ASP-86
OD2
NH1
ARG-89
-2.000
2.000

I

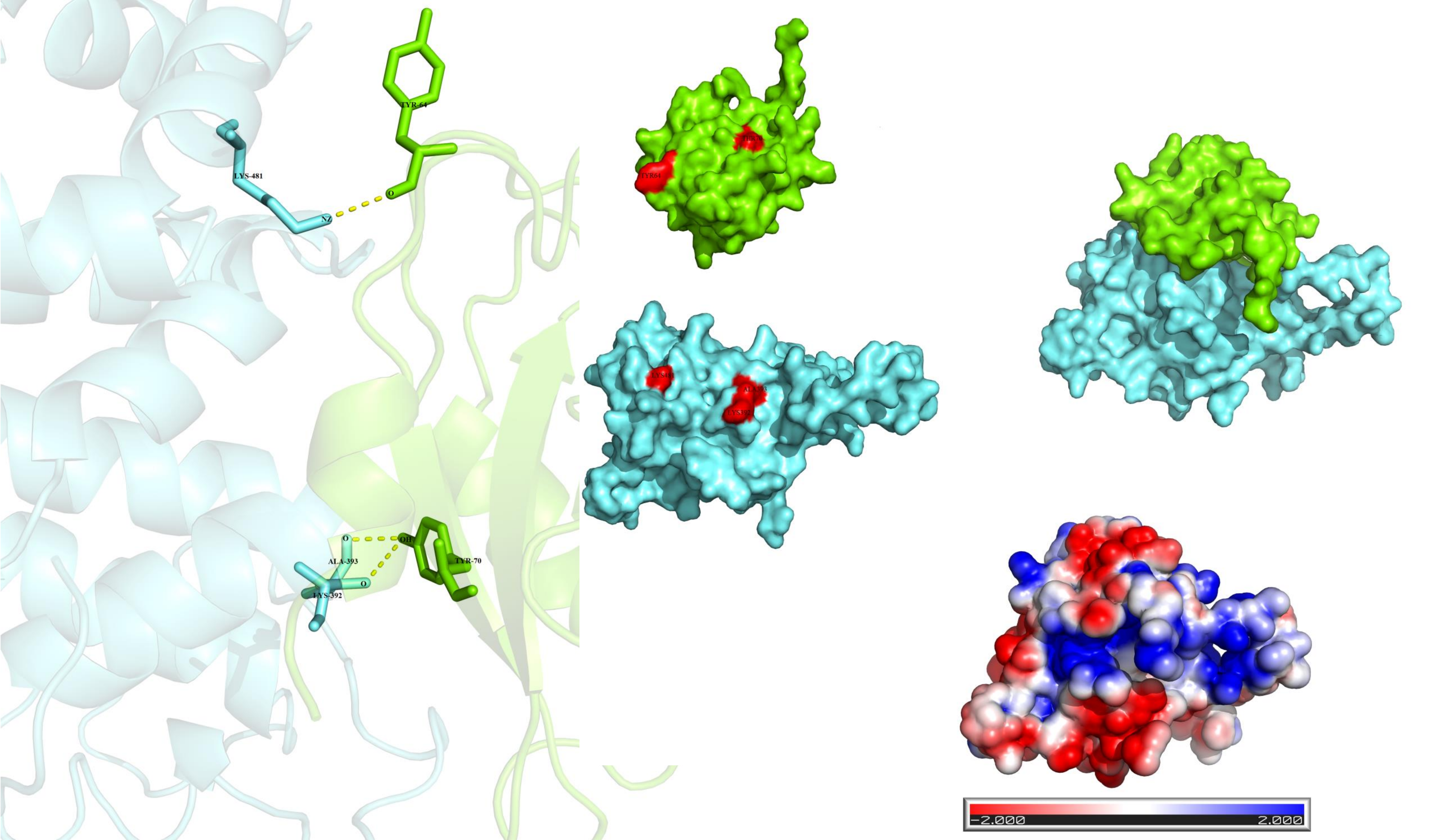

J

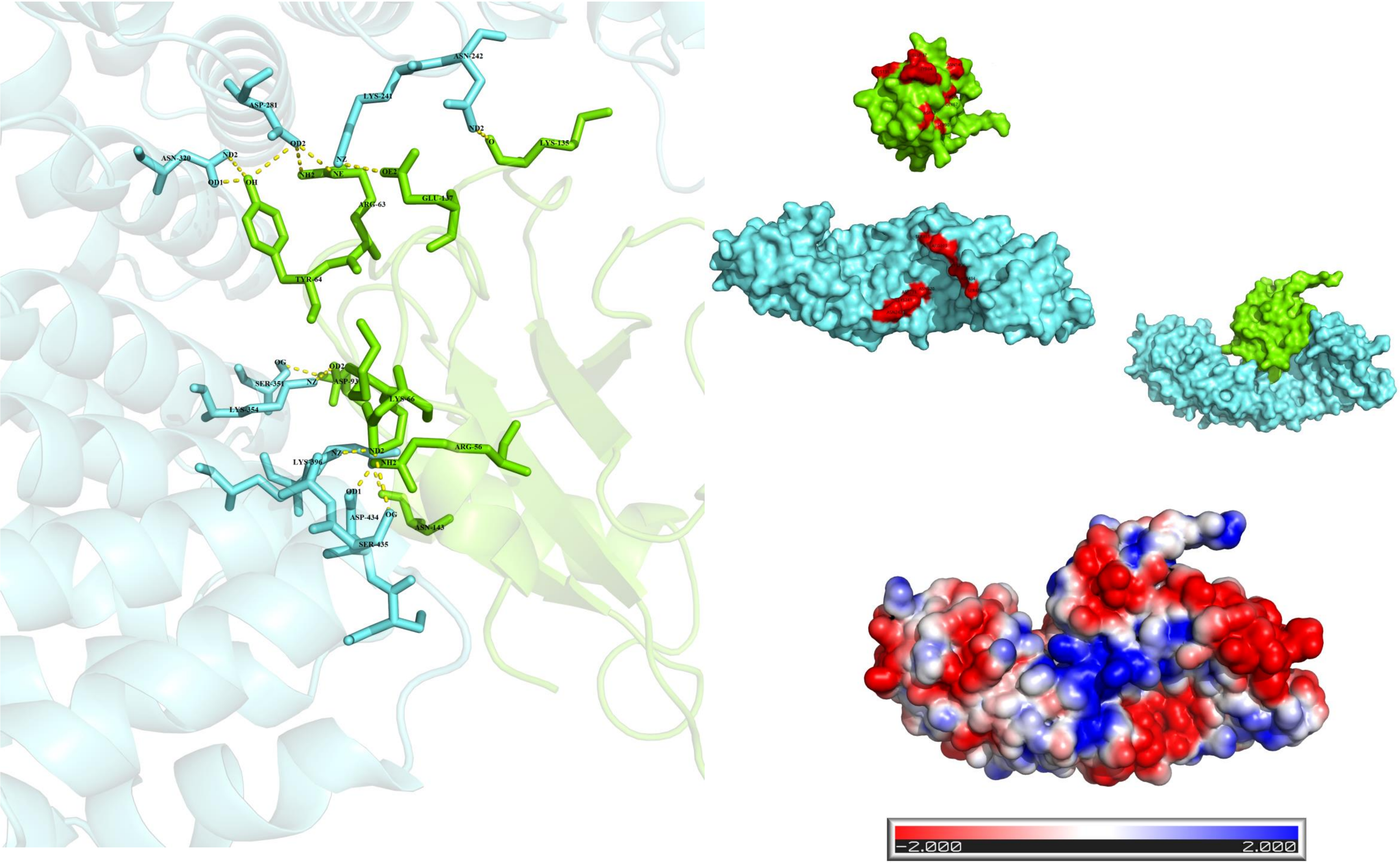
ASN-242
ASP-281
LYS-241
ND2
O
LYS-135
OD2
ASN-320
ND2
NZ
NH2
NE
OE2
OD1
OH
GLU-137
ARG-63
TYR-64
OG
OD2
SER-351
NZ
ASP-93
LYS-66
LYS-354
ARG-56
NZ
ND2
LYS-396
NH2
OD1
ASP-434
OG
ASN-143
SER-435
-2.000
2.000
