# Supplementary material for: Doublecortin undergo nucleocytoplasmic transport via the RanGTPase signaling to promote glioma progression
Source: Cell Commun Signal. 2020 Feb 12;18:24. doi: 10.1186/s12964-019-0485-5 (PMC7017634; doi:10.1186/s12964-019-0485-5)
Supplement: Supplementary file 7 — Additional file 6: Table S1. Comparison of the sequence of recombinant expression vectors of DCX wild type and DCX variant lentiviruses. [file 12964_2019_485_MOESM7_ESM.docx]

Supplementary Table 1

pLenti-CMV-GFP-2A-Puro/DCX-NLS1-deletion（DCXNLS1-del）；

pLenti-CMV-GFP-2A-Puro/DCX-NLS1-mutation（DCXNLS1 mut）；

pLenti-CMV-GFP-2A-Puro/DCX-NLS2-deletion（DCXNLS2-del）；

pLenti-CMV-GFP-2A-Puro/DCX-NLS2-mutation（DCXNLS2-mut）。

**Table 1: Comparison of the sequence of recombinant expression vectors of DCX wild type and DCX variant lentiviruses**

|  | Putative NLS sequence（NLS-1） |
| --- | --- |
| Wild type | GCATCTAGAAATATGAGAGGGTCACGGATGAATGGACTTCCAAGTCCCACT |
| DCXNLS1-del | GCATCTAGAAATATGAGA--------------------------------------------------------------------CTTCCAAGTCCCACT |
| DCXNLS1 mut | GCATCTAGAAATATGAGAGGGTCA*TCA*A*C*GAATGGACTTCCAAGTCCCACT |
| B |  |
|  | Putative NLS sequence（NLS-2） |
| Wild type | CAGGCATTAAGTAATGAGAAGAAGGCCAAGAAGGTACGTTTCTACCGCAAT |
| DCXNLS2-del | CAGGCATTA--------------------------------------------------------------------------------------------------------------------TTCTACCGCAAT |
| DCXNLS2-mut | CAGGCATTAAGTAATGAG*GCAGCA*GCCAAG*GCA*GTAC  GTTTCTACCGCAAT |

DCX wild type and the DCX variant sequence, “-------” indicates missing nucleotides and the oblique body indicates the base mutation.
